# Patient‐Derived 3D‐Bioprinted Intrahepatic Cholangiocarcinoma Models Recapitulate Tumor Autologous Traits and Predict Personalized Adjuvant Therapy

**DOI:** 10.1002/advs.202522025

**Published:** 2026-02-08

**Authors:** Yuce Lu, Liwei Du, Minghao Sun, Kai Zhang, Mingchang Pang, Shangze Jiang, Jiaxun Dong, Xiyue Liu, Bao Jin, Fu Xu, Hang Sun, Jiangang Zhang, Huiyu Yang, Xiaobo Yang, Xin Lu, Yiyao Xu, Haitao Zhao, Shunda Du, Xinting Sang, Yongchang Zheng, Lei Zhang, Xueshuai Wan, Huayu Yang, Yilei Mao

**Affiliations:** ^1^ Department of Liver Surgery Peking Union Medical College (PUMC) Hospital Peking Union Medical College (PUMC) & Chinese Academy of Medical Sciences (CAMS) Beijing China; ^2^ Liver Transplantation Center National Clinical Research Center for Digestive Diseases Beijing Friendship Hospital Capital Medical University Beijing China; ^3^ Department of Head and Neck Surgery National Cancer Center/National Clinical Research Center for Cancer/Cancer Hospital Chinese Academy of Medical Sciences and Peking Union Medical College Beijing P. R. China; ^4^ Department of Neurosurgery Xuanwu Hospital Capital Medical University Beijing P.R. China

**Keywords:** 3D bioprinting, drug sensitivity prediction model, intrahepatic cholangiocarcinoma, personalized and precision medicine

## Abstract

Intrahepatic cholangiocarcinoma (ICC) is a highly aggressive malignancy with a dismal prognosis, and pronounced interpatient heterogeneity severely limits the efficacy of systemic therapies, underscoring the need for rapid and accurate functional platforms to guide individualized drug selection. Here, we develop a clinically oriented, patient‐derived, 3D bioprinted in vitro model for personalized drug sensitivity assessment in ICC. Using a compositionally defined and cost‐effective GelMA/HAMA composite hydrogel, we reconstruct a tumor microenvironment that supports rapid self‐organization and sustained viability of primary ICC cells. Histological analyses, marker expression profiling, and bright‐field imaging demonstrate close similarity to matched patient tumor tissues. Genomic and transcriptomic fidelity are further confirmed by whole‐exome and RNA sequencing, revealing preserved driver mutations and transcriptional programs. Drug sensitivity testing was performed on tumor samples from 21 ICC patients using clinically relevant agents. Notably, in patients receiving neoadjuvant therapy, in vitro drug responses were fully consistent with clinical outcomes. Longitudinal follow‐up further showed that recurrence occurred exclusively in patients who did not receive the predicted sensitive therapies. Importantly, clinically actionable drug response profiles were generated within 10 days. Collectively, this platform provides a rapid, reproducible, and patient‐specific functional drug testing strategy with strong potential for clinical translation.

## Graphical abstract

Leveraging 3D bioprinting, this study establishes patient‐derived in vitro models of intrahepatic cholangiocarcinoma. These models faithfully recapitulate the histopathology, molecular profiles, and genomic characteristics of the original patient tumors. Importantly, the engineered models demonstrate a strong correlation with clinical outcomes of the corresponding patients, offering a highly representative platform for translational oncology research.

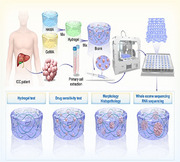

## 1 Introduction

Intrahepatic cholangiocarcinoma (ICC) is the second most common primary liver cancer, accounting for approximately 20% of hepatic malignancies and 3% of gastrointestinal cancers, and is associated with a dismal prognosis due to its insidious onset and late‐stage diagnosis [1]. The five‐year survival rate of ICC remains as low as 9%, and although surgical resection offers the only potentially curative option, postoperative recurrence is frequent, with five‐year survival rates of only 20%–40% [2]. For patients with unresectable or advanced ICC, systemic therapies such as chemotherapy, targeted therapy, and immunotherapy have demonstrated survival benefits [3]. Gemcitabine plus cisplatin remains the standard first‐line regimen, and the addition of immune checkpoint inhibitors such as pembrolizumab has further improved overall survival [4, 5]. Moreover, neoadjuvant chemotherapy has been shown to facilitate tumor downstaging and reduce postoperative mortality. Collectively, these advances highlight that appropriate drug selection is critical for improving clinical outcomes [6, 7, 8, 9]. However, pronounced interpatient heterogeneity in ICC leads to highly variable therapeutic responses [10], underscoring the urgent need for accurate, rapid, and cost‐effective platforms to guide individualized treatment decisions.

To establish in vitro models of ICC that can effectively support clinical decision‐making, an ideal system should be cost‐effective, highly reproducible, amenable to automation, efficient to construct, and capable of faithfully recapitulating the tumor microenvironment, while enabling drug sensitivity assessment within a clinically relevant timeframe. However, most current patient‐derived in vitro drug sensitivity platforms for ICC still rely on patient‐derived xenograft (PDX) models or Matrigel‐based organoid systems, both of which face substantial barriers to clinical translation. First, the establishment of PDX models typically requires 4–8 months [11], whereas the median survival of patients with advanced biliary tract cancer is less than one year, severely limiting their utility for timely, personalized therapeutic guidance [12]. In addition, both animal models and Matrigel‐based systems are associated with high economic costs, which restrict their scalability and broader clinical translation. More importantly, organoid models cultured in Matrigel tend to progressively lose non‐tumor components of the tumor microenvironment during long‐term passaging [13]. In the context of ICC, multiple studies have demonstrated that cancer‐associated fibroblasts, immune cells, endothelial cells, and neural‐associated cells play critical roles in regulating tumor invasiveness, metastatic potential, and therapeutic resistance [14, 15, 16]. The loss of these non‐tumor components therefore compromises the ability of existing models to accurately predict patient‐specific drug responses.

To date, only one study has applied 3D bioprinting technology to ICC, in which an in vitro model was constructed using a composite hydrogel composed of gelatin, alginate, and Matrigel [17]. However, this study presents several critical limitations. First, the reliance on Matrigel inevitably introduces challenges related to its high cost, substantial batch‐to‐batch variability, and undefined composition, thereby limiting model reproducibility and clinical translatability. Second, the study evaluated only a single therapeutic agent, which is insufficient to support personalized treatment strategies and fails to capture the pronounced intertumoral heterogeneity and complex drug resistance mechanisms commonly observed in ICC. Single‐drug screening not only restricts the assessment of combination or alternative therapeutic regimens but also compromises the reliability of predicting clinical treatment responses. Finally, the stability of this model in larger patient cohorts, as well as its concordance with actual clinical outcomes, has yet to be systematically validated. Consequently, developing cost‐effective in vitro ICC models that more faithfully recapitulate the tumor microenvironment while enabling rapid, multi‐drug sensitivity assessment remains a critical unmet need in the field.

Building upon these limitations, we developed a clinically oriented, patient‐derived 3D bioprinted in vitro model to support therapeutic decision‐making for ICC. To effectively recapitulate the ICC tumor microenvironment, we selected commercially available, cost‐effective, and compositionally defined gelatin methacryloyl (GelMA) as the primary extracellular matrix component. On this basis, we further incorporated methacrylated hyaluronic acid (HAMA) as a complementary matrix component. Hyaluronic acid is a major constituent of the native extracellular matrix and possesses excellent biocompatibility and viscoelasticity [18, 19]. When combined with collagen‐derived materials, it can significantly enhance the mechanical strength and porosity of hydrogels, making such composites more suitable for modeling tumors with high matrix stiffness [20]. Such GelMA‐HAMA composite hydrogels have previously been applied to model tumors with high matrix stiffness [21]. Given that ICC typically exhibits a stiff, stroma‐rich tumor phenotype, we ultimately selected a GelMA‐HAMA composite as the extracellular matrix for the in vitro 3D bioprinted ICC model, thereby enabling a more faithful reconstruction of its tumor microenvironment.

In this study, we systematically optimized a bioink formulation tailored for ICC and successfully established a 3D bioprinted model for patient‐derived ICC drug sensitivity testing using a composition of 6.5% GelMA and 0.5% HAMA. The resulting constructs rapidly self‐organized into complex architectures in vitro while preserving key histological and molecular pathological characteristics. Whole‐exome sequencing and RNA sequencing further confirmed the high fidelity of the model to the corresponding primary tumors. Importantly, this platform enables multi‐drug sensitivity assessment within 10 days, with drug response profiles showing strong concordance with patients’ clinical outcomes. Collectively, our approach provides a rapid, cost‐effective, personalized, and accurate in vitro system to support therapeutic decision‐making for patients with ICC.

## 2 Results

### 2.1 Bioink Selection and 3D Bioprinting Workflow

Bioink typically consists of a hydrogel as an extracellular matrix and cell components embedded within the hydrogel. The hydrogel serves not only as a three‐dimensional (3D) structural scaffold for cells but also regulates mass exchange between cells and their microenvironment while maintaining phenotypic stability [22, 23]. Among the available biomaterials, GelMA has been widely used in in vitro three‐dimensional bioprinting owing to its well‐defined composition and excellent biocompatibility. HAMA, a key component of the extracellular matrix, has also been extensively employed for the construction of in vitro disease models. It plays essential roles in promoting clonal cell expansion, supporting tissue regeneration, and enabling drug screening applications [24, 25, 26, 27].

**FIGURE 1:**
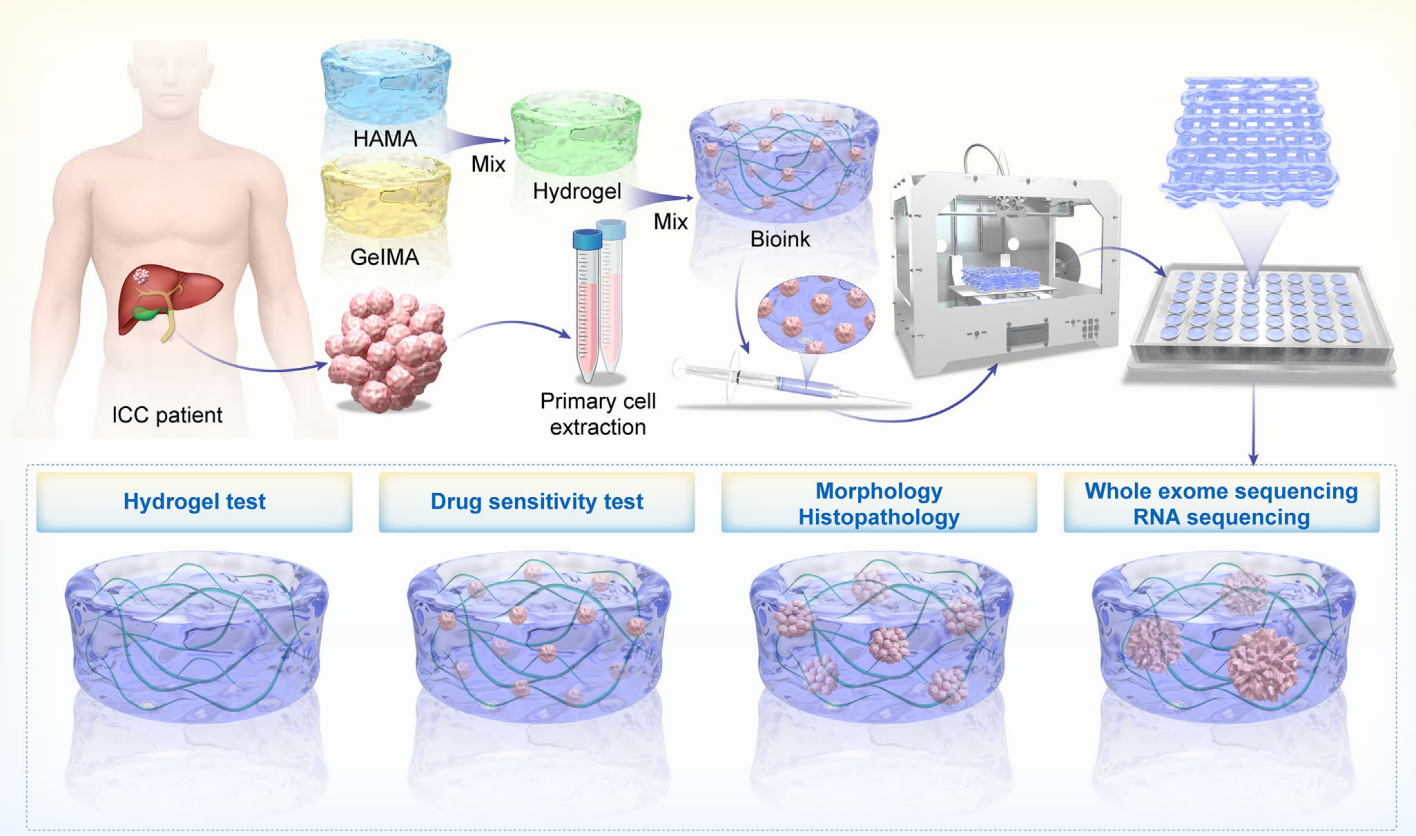
Workflow for patient‐derived ICC_3DP construction and validation for clinical drug response prediction.

Substantial studies have confirmed that the GelMA/HAMA composite hydrogel system provides an ideal three‐dimensional microenvironment, which supports high‐efficiency clonal expansion of diverse cell types while maintaining their phenotypic stability. This system has also demonstrated strong predictive capability in multiple drug screening assays. Furthermore, the combination of these biomaterials exhibits remarkable regenerative capacity and excellent biocompatibility in vivo, accompanied by a significantly reduced foreign body reaction compared to single‐component hydrogels [28, 29, 30, 31]. Building upon prior research, this study systematically evaluates GelMA/HAMA composite ratios to optimize a bioink formulation tailored for ICC in vitro modeling. By refining both the material composition and bioprinting workflow (Figure 1), we aim to construct a TME that closely recapitulates in vivo conditions, thereby enhancing the reliability of drug response assays and accuracy of mechanistic studies.

### 2.2 Rheological Property Assessment, Microstructural Scanning, and Biocompatibility Evaluation of Bioinks

**FIGURE 2:**
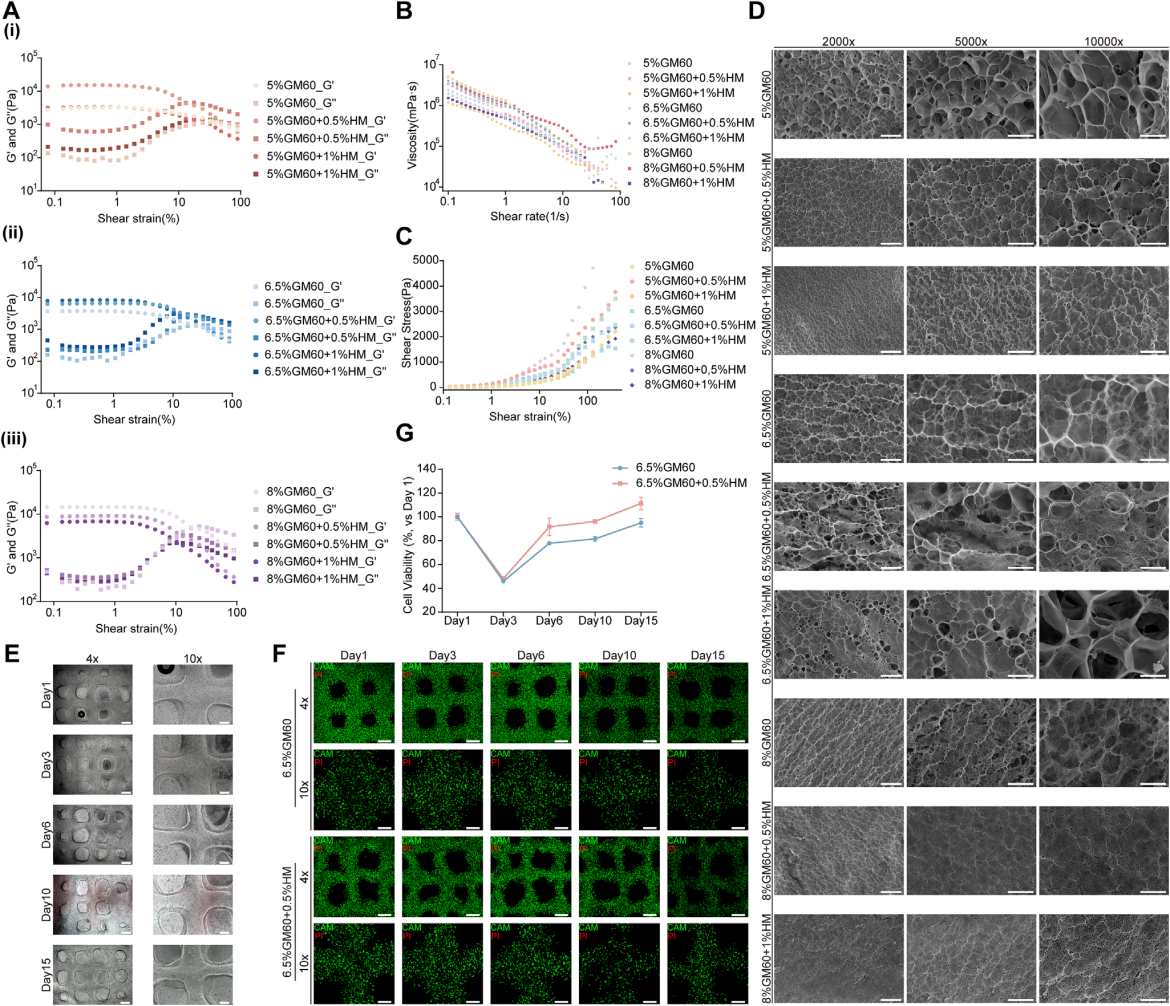
Rheological properties, microstructural characteristics, and biocompatibility of GelMA/HAMA bioinks. (A) Representative compression curves of hydrogels composed of (i) 5%, (ii) 6.5%, and (iii) 8% GelMA blended with varying HAMA concentrations (0%–1%), tested at 37°C. (B) Viscosity and shear‐thinning behavior of different hydrogel systems at 37°C. (C) Typical compressive stress‐strain profiles of hydrogels with varying GelMA/HAMA ratios. (D) Cryo‐SEM images illustrating the porous microstructure of nine GelMA/HAMA hydrogels at different concentration ratios.(2000× scale bar: 100 µm; 5000×: 50 µm; 10 000×: 25 µm). (E) Bright‐field images demonstrating the stable 3D architecture of RBE‐laden 6.5% GelMA bioink constructs after 15 days in culture (left scale bar: 500 µm; right: 200 µm). (F) Live/dead assay (Calcein‐AM/PI staining) of RBE cells (5×10^6^ cells/mL) embedded in bioinks at days 1, 3, 6, 10, and 15 post‐printing (green: live; red: dead; 4× scale bar: 500 µm; 10×: 200 µm). (G) Proliferation kinetics of RBE cells encapsulated in two selected hydrogel formulations over 15 days.

Rheological characterization revealed significant mechanical behavior differences among bioinks with varying GelMA/HAMA ratios (Figure 2A). Increasing GelMA concentration systematically elevated the hydrogel storage modulus (*G*′), indicating enhanced crosslinking network density. Notably, higher HAMA content synergistically amplified the elastic modulus, likely attributable to molecular interactions between GelMA and HAMA that fostered a more robust 3D network architecture. All formulations exhibited characteristic gel‐sol transition behavior while maintaining predominant elastic responses (*G*′ > *G*″) under high shear stress, demonstrating exceptional structural recoverability. This property is critical for ensuring extrusion stability during bioprinting processes.

Shear rate sweep experiments systematically evaluated the shear‐thinning behavior of bioinks with varying compositions. The results demonstrated a significant decrease in apparent viscosity with increasing shear rates (Figure 2B). This rheological profile suggests that the bioinks retained high viscosity under low shear conditions, which supports structural stability during printing, while their viscosity decreased at higher shear rates, allowing smooth extrusion through the nozzle. Strain amplitude testing further showed that bioinks containing 0.5% HAMA exhibited greater stress tolerance at comparable strain levels, reflecting improved mechanical resilience suitable for precise layer‐by‐layer deposition (Figure 2C).

The porous architecture of hydrogels serves as a critical foundation for cell attachment, with its optimized microstructure directly influencing nutrient exchange, cellular communication, and signal transduction processes. Using cryo‐scanning electron microscopy, we characterized the microstructures of bioinks with different compositions, revealing that HAMA‐incorporated bioinks exhibited denser pore packing configurations, which may effectively reduce cell loss during culture (Figure 2D). Notably, when HAMA concentration reached 1%, the hydrogel pore size became excessively small, not only hindering cellular migration into the pores but also compromising the exchange efficiency of nutrients and metabolic byproducts.

Given that the in vitro 3D printing process may cause certain cellular damage and different bioink formulations could potentially influence cell viability, we conducted a 15‐day culture experiment using RBE cells encapsulated in two bioink formulations: 6.5% GelMA and 6.5% GelMA/0.5% HAMA. Cellular behavior was assessed on days 1, 3, 6, 10, and 15 via optical microscopy, calcein‐AM/propidium iodide (CAM/PI) live‐dead staining, and CellTiter 3D viability assays. The results demonstrated that both bioinks maintained structural integrity throughout the 15‐day culture period, with the HAMA‐containing system exhibiting reduced cell leakage (Figure 2E; Figure S1A). Live‐dead staining confirmed excellent viability and minimal cell mortality in both groups (Figure 2F). Cell proliferation assays revealed a transient decline in viability at day 3, followed by stabilization from day 6 onward, with cells in the HAMA‐supplemented bioink displaying higher metabolic activity (Figure 2G).

Considering the relatively stiff nature of intrahepatic cholangiocarcinoma tissues, together with prior evidence that gelatin/HAMA composites provide favorable biocompatibility and viscoelastic properties [20], and supported by our rheological measurements and ultrastructural observations, we selected a composite bioink consisting of 6.5% GelMA, 0.5% HAMA, and 0.125% LAP for ICC bioprinting.

### 2.3 Generation and Characterization of Patient‐derived ICC_3DP Models in Vitro

This study initially enrolled 40 patients who underwent radical tumor resection at Peking Union Medical College Hospital. However, some patients were excluded following histopathological confirmation of non‐intrahepatic cholangiocarcinoma. Three cases were further eliminated due to insufficient tissue quantity for subsequent experiments, though all samples remained microbiologically uncontaminated. Owing to limitations in primary cell yield, whole‐exome sequencing (WES) was performed on samples from 6 patients; RNA sequencing was conducted for 4 patients, while drug sensitivity testing was conducted on specimens from 21 patients. Patient characteristics are detailed in Table S1. All constructs were fabricated on a precision 3D bioprinting apparatus, ensuring consistent temperature regulation and aseptic operation throughout the process.

### 2.4 The ICC_3DP Model Successfully Retained the Histopathological Characteristics of the Parental Tumor In Vitro

**FIGURE 3:**
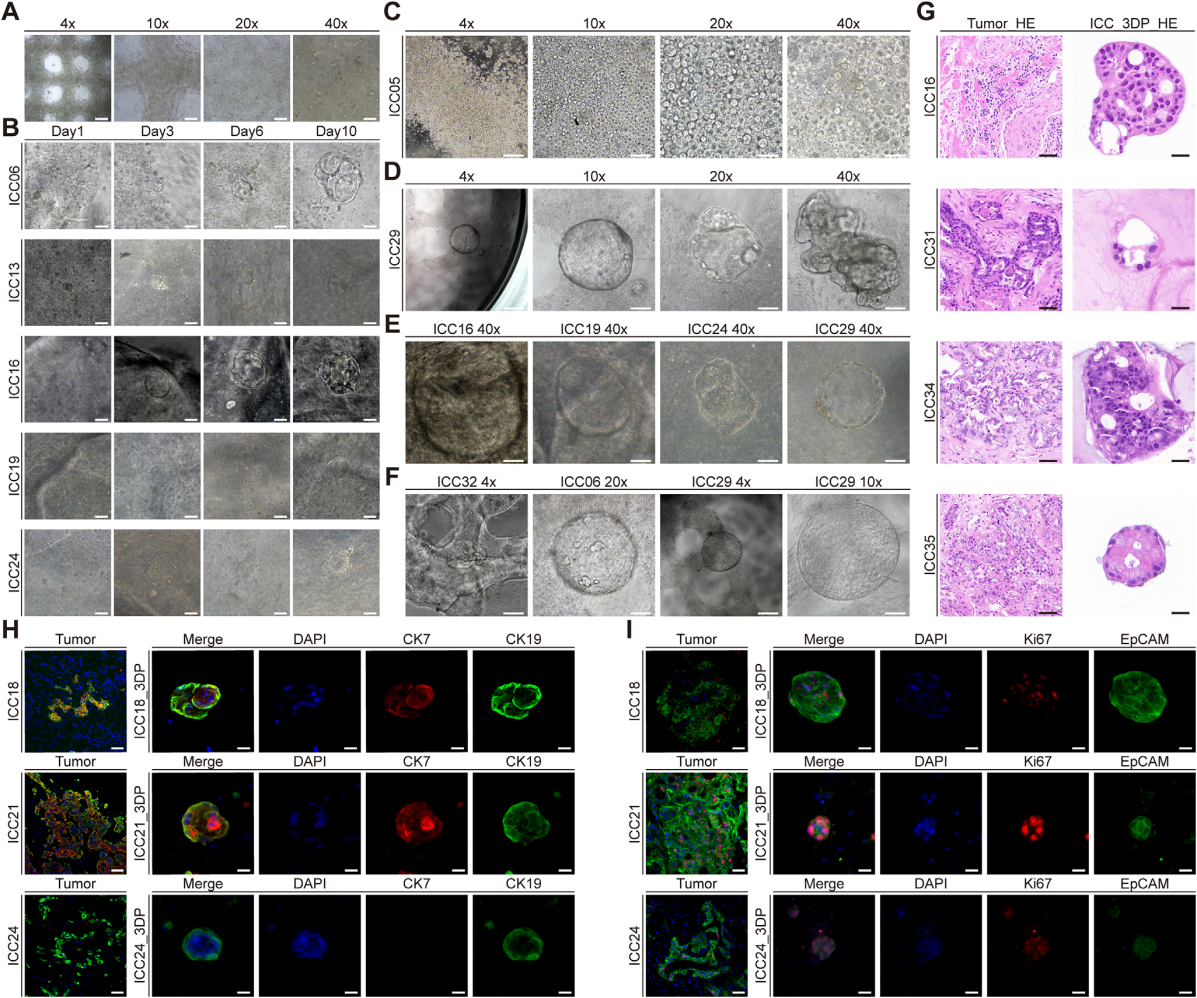
ICC_3DP preserves morphological, histological, and molecular characteristics of parental tumors. (A) Bright‐field images of patient‐derived tumor cells on day 1 post‐bioprinting (scale bars from left: 500, 200, 100, 50 µm). (B) Patient‐derived primary cells self‐assemble into organoid‐like structures with complex architecture by day 10 (scale bar: 50 µm). (C,D) Morphological presentation of ICC05 (C) and ICC29 (D) bioprinted constructs at various magnifications, showing spherical cell aggregation and prominent tumor cell clusters with 3D organization (scale bars from left: 500, 200, 100, 50 µm). (E) Representative bright‐field images of bioprinted constructs from four patients (scale bar: 50 µm). (F) Organoid‐like morphology across different patients at low magnification (4× scale bar: 500 µm; 10×: 200 µm; 20×: 100 µm). (G) H&E staining demonstrating conserved histological features between ICC_3DP and matched parental tumor tissues (left scale bar: 50 µm; right scale bar: 20 µm). (H) Immunofluorescence co‐staining of cholangiocarcinoma markers (CK7, CK19) in parental tumors and ICC_3DP models (scale bars: 40 µm for parental tumors; 20 µm for 3DP models). (I) Immunofluorescence co‐staining of cholangiocarcinoma markers (Ki67, EpCAM) in parental tumors and ICC_3DP models (scale bars: 40 µm for parental tumors; 20 µm for 3DP models).

Considering the small size and dense texture characteristic of ICC tissue, we optimized the primary cell extraction protocol and correspondingly adjusted the bioprinting parameters and workflow. Figure 3A displays the morphological characteristics of the 3DP model under different magnifications on the first day post‐printing. The images demonstrate appropriate cell density within the hydrogels, facilitating cell growth, cell‐cell interactions, and cluster formation. During in vitro culture, the primary intrahepatic cholangiocarcinoma cells exhibited excellent proliferation capacity within the hydrogel matrix. The cells progressively aggregated from initially dispersed single cells into spheroids, ultimately forming typical organoid‐like structures by day 10 in most cultures (Figure 3B; Figure S1B). By day 10, the bioprinted model developed complex three‐dimensional architectures that could not be achieved under conventional two‐dimensional culture conditions, indicating excellent biocompatibility of the bioink with primary intrahepatic cholangiocarcinoma cells (Figure S2A). Notably, tumor cells derived from different patients displayed significant growth heterogeneity (Figure S2B). For instance, ICC05_3DP developed tightly packed cellular spheroids within just 7 days of culture, with minimal observable single‐cell dispersion—compelling evidence validating the exceptional performance of our engineered bioink (Figure 3C; Video S1). A critical methodological advancement was our complete substitution of conventional Matrigel with chemically defined commercial hydrogels throughout this study. Particularly striking was the ICC29 case, where primary tumor cells autonomously organized into distinct organoid structures readily visible at low‐power magnification, while high‐resolution imaging comprehensively unveiled their sophisticated 3D microarchitectural features (Figure 3D; Videos S4–S8). With prolonged culture, these in vitro models progressively developed morphological characteristics resembling a paired in vivo tumor. As demonstrated by the ICC16_3DP, ICC19_3DP, and ICC24_3DP samples, high‐magnification imaging revealed densely packed cellular spheroids occupying nearly the entire field of view. Strikingly, in the ICC32, ICC06, and ICC29 samples, the resulting spheroids were morphologically comparable to those formed in previous studies using Matrigel‐based in vitro cultures of intrahepatic cholangiocarcinoma [32, 33], with some cells even exceeding the encapsulation boundary of the hydrogel matrix (Figures 3E, F; Videos S2–S8).

We utilized H&E staining to evaluate the histological fidelity of the 3D‐printed models. The analysis demonstrated that the ICC_3DP constructs successfully recapitulated the cellular heterogeneity of intrahepatic cholangiocarcinoma, thereby maintaining acinar characteristics that closely resembled the original tumor (Figure 3G; Figure S2C).

To further validate the pathological fidelity of our 3D‐printed models compared to parental tumors, we conducted paired immunofluorescence analysis of cholangiocarcinoma markers (CK7, CK19), proliferative marker Ki67, and invasion marker EpCAM. In CK7/CK19‐positive cases (ICC18 and ICC21), the printed constructs exhibited staining patterns identical to those of the original tissues. In contrast, CK7‐negative ICC24 models faithfully reproduced the phenotype of the parental tumor. (Figures 3H, I). Notably, all tested cases (ICC18, ICC21, ICC24) maintained high concordance in both Ki67 and EpCAM expression between tumors and their corresponding models. These findings conclusively demonstrate that our 3DPs accurately preserve key molecular characteristics of parental tumors and exhibit superior pathological recapitulation across diverse patient‐derived samples.

**FIGURE 4:**
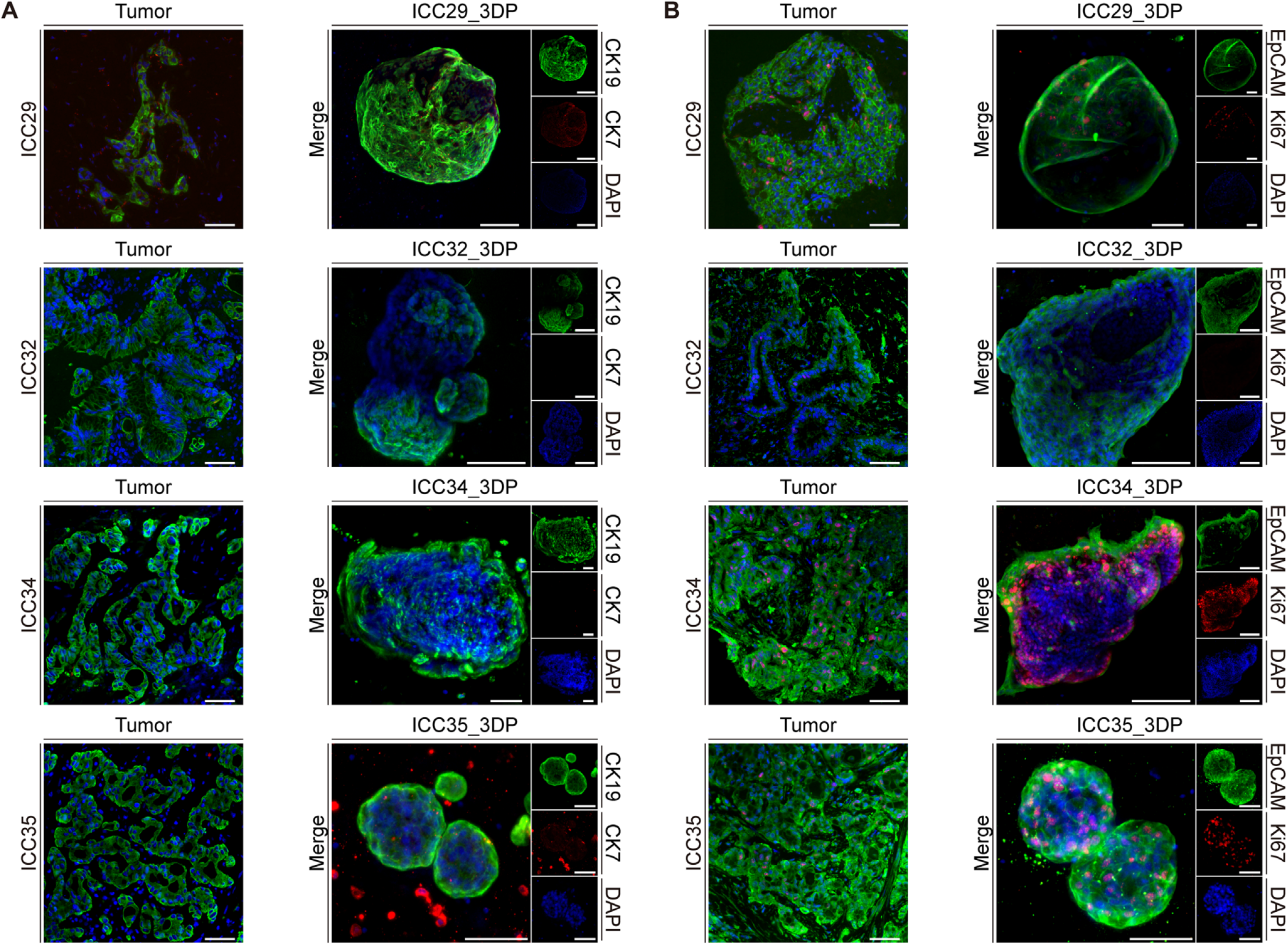
ICC_3DP maintains morphological polarity and molecular signatures of parental tumors. (A) Immunofluorescence staining of ICC markers CK7 (red) and CK19 (green) demonstrating conserved expression patterns between ICC_3DP models and matched parental tumor tissues (scale bar: 50 µm). (B) Proliferation marker Ki67 (red) and epithelial cell adhesion molecule EpCAM (green) show comparable distribution in ICC_3DP constructs and original tumors, confirming preservation of tumor proliferation characteristics (scale bar: 50 µm).

We systematically observed the growth dynamics of isolated single cells and small cell clusters derived from primary tumor tissues within the GelMA‐HAMA hydrogel system. Remarkably, these cells demonstrated exceptional proliferative capacity, particularly in samples from ICC29, ICC32, ICC34, and ICC35 patients, where primary tumor cells spontaneously organized into intricate three‐dimensional architectures visible under low magnification microscopy (Figures 4A, B; Figure S3A, B). Notably, cell clusters from ICC32 and ICC34 specimens exhibited distinct polarization characteristics. Cells expressing EpCAM were predominantly localized at the periphery, a pattern scarcely observed in the central region of the cell clusters. A similar distribution was noted for Ki67‐positive cells. This spatial arrangement reflects a transition from a low differentiation state to a functional phenotype, indicative of cellular polarization and functional maturation, which aligns with earlier reports [34]. These observations suggest that the clusters expand outward through proliferation, while cells at the periphery gradually lose their proliferative capacity and acquire functional characteristics. This phenomenon may mimic the process by which tumor cells develop invasive potential and functional specialization during division. This three‐dimensional culture system not only recapitulated the proliferation‐dominant growth pattern of tumor cells but also progressively developed functionally compartmentalized tissue structures during later culture stages, closely mimicking the native in vivo tumor microenvironment. These findings establish a more physiologically relevant research model for conducting precision diagnostics based on patient‐specific tumor characteristics.

### 2.5 ICC_3DP Models Retained Key Genetic Mutations and Intratumoral Heterogeneity of Their Parental Tumors

**FIGURE 5:**
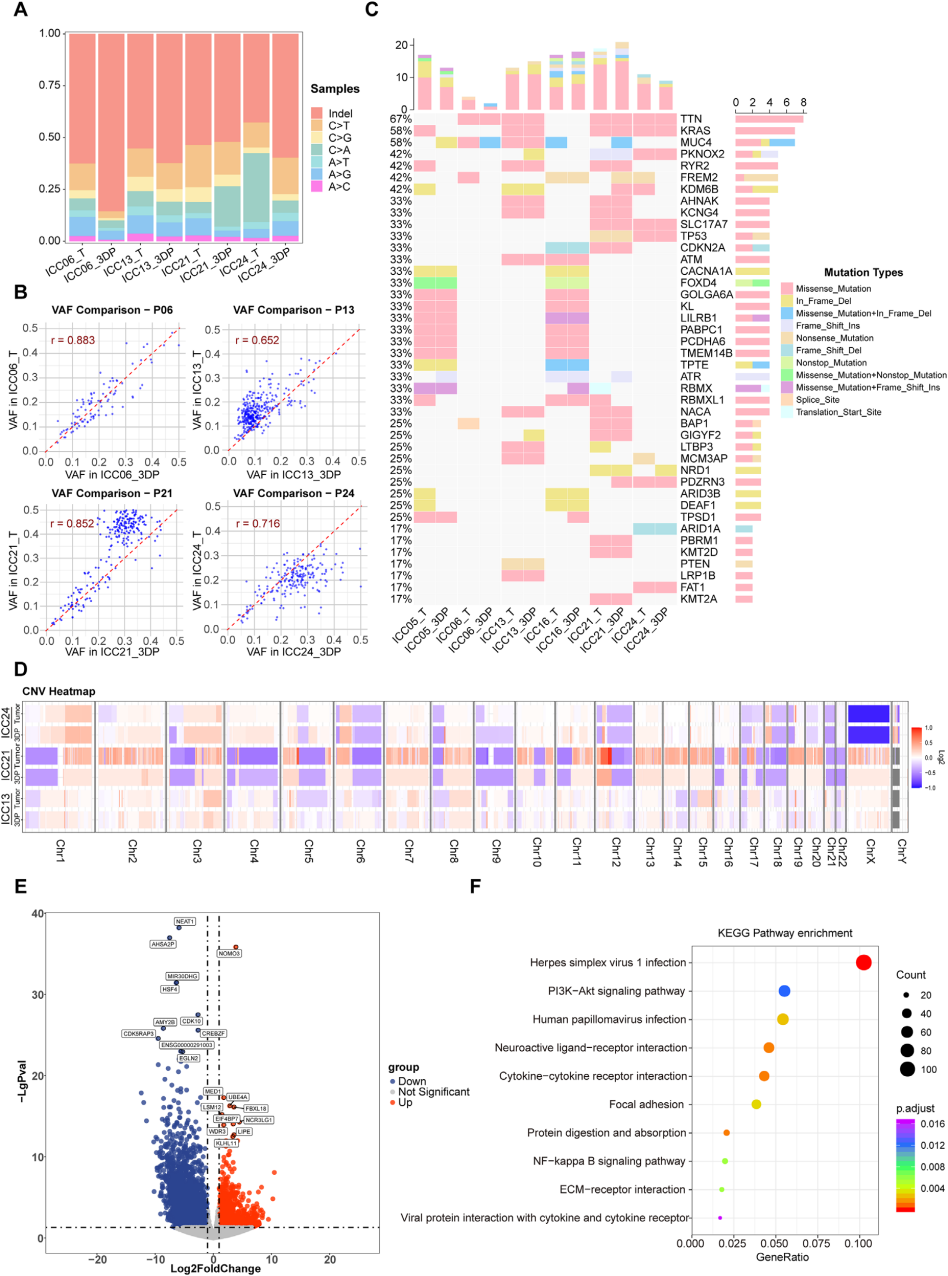
Multi‐omics landscape of ICC_3DP models and matched parental tumors. (A) The proportion of exon variations in four pairs of paired samples. (B) Correlation of DNA variant allele frequency (VAF) between ICC_3DP models and their parental tumors. The linear regression fit (red line) and correlation coefficient (r) are shown. (C) Oncoplot of frequently mutated somatic driver genes in parental tumors and their derived ICC_3DP models (T, tumor; P, ICC_3DP). (D) Whole‐genome copy number variation (CNV) profiles of ICC_3DP models and original tumors from three representative patients (ICC13, ICC21, ICC24). (E) Volcano plot identifying differentially expressed genes (DEGs) between ICC_3DP models and parental tumors. (F) KEGG pathway enrichment analysis of differentially expressed genes between the ICC_3DP model and the corresponding parental tumor.

To assess the genomic concordance between ICC_3DP models and the parental tumors, we performed WES on matched 3DP models (maintained in vitro for over 10 days) and primary tumor samples. WES analysis of six matched pairs revealed that the ICC_3DP models largely retained the spectrum of single‐nucleotide variants (SNVs) present in the original tumors. We observed a high concordance in the profiles of SNVs, insertions, and deletions (indels) between the models and the source tissues (Figure 5A). Furthermore, comparison of the variant allele frequencies demonstrated a strong correlation, indicating that our in vitro model effectively preserves both tumor heterogeneity and purity (Figure 5B).

Integration of our sequencing data and published evidence enabled the comprehensive characterization of a recurrent ICC mutational profile, encompassing over 40 significantly mutated genes and their SNV landscapes [35, 36, 37]. In our models, TTN, KRAS, and MUC4 were the most frequently mutated genes, and the variant frequencies of all key drivers remained stable. The driver spectrum concurred with established literature, encompassing TP53, KRAS, FGFR2, and IDH1/2 mutations, BAP1 fusions, and ARID1A loss (Figure 5C) [38, 39]. Critically, perfect (100%) mutational concordance was achieved for TP53, PTEN [40, 41, 42], ARID1A, and CDKN2A across all matched pairs, extending to PBRM1 [43] and AHNAK. Other key genes (BAP1, KMT2D, ARID1B, RYR2) showed high retention (>80%). CNV analysis demonstrated that ICC_3DPs and their matched parental tumors share highly similar patterns of genomic losses and gains (Figure 5D). This comprehensive molecular validation establishes the ICC_3DP platform as a highly faithful model for preserving patient‐specific tumor genetics, providing a reliable tool for personalized oncology studies.

Furthermore, comparative transcriptomic analysis was performed between the ICC_3DP model and its matched primary tumor specimen to elucidate key pathways and genes driving tumorigenesis. This analysis identified numerous differentially expressed genes (DEGs) in the 3DP model relative to the parental tumor (Figure 5E). Notably, the ICC_3DP model exhibited significant upregulation of NOMO3, FBXL18, and UBAE compared to the original tumor [44]. These genes have been implicated in various malignancies and are known to contribute to extracellular matrix (ECM) remodeling, proteasomal degradation, tumor proliferation, and invasion.

KEGG pathway enrichment analysis of the DEGs between the ICC_3DP model and the corresponding parental tumor further revealed significant alterations in the PI3K/AKT and ECM‐receptor interaction signaling pathways (Figure 5F). These pathways are closely associated with ICC proliferation, invasion, and drug resistance [45]. The findings suggest that the ECM components derived from the bioink may actively promote malignant behaviors in primary ICC cells.

### 2.6 ICC_3DP Models Faithfully Recapitulate Patient‐Specific Drug Response Profiles

Based on previous studies, drug sensitivity testing using 3D bioprinted primary tumor models typically requires a minimum cell density of approximately 3 × 10^6^ to 6 × 10^6^ cells/mL [29]. To assess whether variation in initial cell density within this range could confound downstream analyses, bioinks containing two different initial cell concentrations (3 × 10^6^ and 6 × 10^6^ cells/mL) were evaluated using tumor samples obtained from two independent ICC patients. Live/dead staining was performed on ICC40_3DP constructs generated at both cell densities on days 1, 3, 6, and 10 after printing. Both conditions exhibited consistently high overall cell viability throughout the culture period (Figure S4A). Quantitative analysis, performed using ImageJ by counting live and dead cells from three randomly selected fields of view, revealed a modest reduction in viability in the lower‐density constructs at day 3. Notably, this difference was transient and was no longer observed at later time points, with comparable viability levels between the two groups on days 6 and 10 (Figure S4B). Bright‐field imaging of ICC39_3DP constructs further demonstrated that models generated at both cell densities followed similar overall growth trajectories during the culture period (Figure S4C). Constructs printed with the higher initial cell density exhibited a more pronounced tendency toward early cell aggregation and formation of larger spheroidal structures, whereas lower‐density constructs showed slightly delayed clustering during the early culture phase. Importantly, by the predefined time point for drug treatment initiation (day 7 post‐printing), constructs from both conditions displayed comparable structural organization and viability, supporting standardized timing for subsequent drug sensitivity testing.

To directly determine whether initial cell density influenced drug sensitivity readouts, parallel drug response assays were performed using constructs printed at both cell concentrations. Across four clinically relevant agents (gemcitabine, cisplatin, oxaliplatin, and lenvatinib), dose‐response curves were highly comparable between the two density conditions in both ICC39 and ICC40 models (Figure S5A,B). Corresponding IC_50_ values showed no systematic differences between 3DP models generated at 3 × 10^6^ and 6 × 10^6^ cells/mL (Figure S5C,D). Although minor differences in cell viability were observed at isolated drug concentrations, particularly at intermediate or near‐saturating doses, these localized variations did not alter the overall dose‐response relationships or the final interpretation of drug sensitivity.

Collectively, these results demonstrate that within the tested range, variation in initial cell density does not compromise construct standardization, culture timeline, or the robustness and comparability of drug sensitivity measurements. To ensure stable model establishment across heterogeneous patient samples, particularly those with intrinsically low post‐isolation viability, a moderately higher initial cell density of approximately 5 × 10^6^ cells/mL was selected for subsequent experiments.

To evaluate whether ICC spheroids generated by 3D bioprinting exhibit comparable morphological and functional characteristics to conventional Matrigel‐based organoids, we performed parallel cultures using primary tumor cells from two ICC patients (ICC36 and ICC37).

Bright‐field imaging revealed that spheroids formed in the 3D‐printed constructs displayed morphology highly similar to Matrigel‐cultured counterparts, including compact cellular aggregation and spherical architecture (Figure S6A). No apparent differences were observed in spheroid integrity or consistency between the two culture systems during the observation period.

We next conducted side‐by‐side drug sensitivity testing using both platforms. Dose‐response analyses demonstrated highly comparable drug response profiles between 3D‐printed spheroids and Matrigel organoids across multiple agents, including gemcitabine, cisplatin, oxaliplatin, and lenvatinib, in both ICC36 and ICC37 models (Figure S6B,C). IC_50_ values derived from the two systems were closely aligned for all tested drugs.

At individual concentration points, minor differences in cell viability were occasionally observed (Figure S6D,E). However, aggregated analyses using violin plots showed no statistically significant differences in overall viability distributions between the 3D‐printed and Matrigel groups for any drug tested (Figure S6F,G). Importantly, these localized variations did not result in meaningful differences in IC_50_ or overall drug sensitivity trends.

Collectively, these results demonstrate that ICC spheroids generated by 3D bioprinting exhibit morphological and functional parity with Matrigel‐cultured organoids, supporting the validity of the ICC_3DP platform for drug sensitivity testing.

**FIGURE 6:**
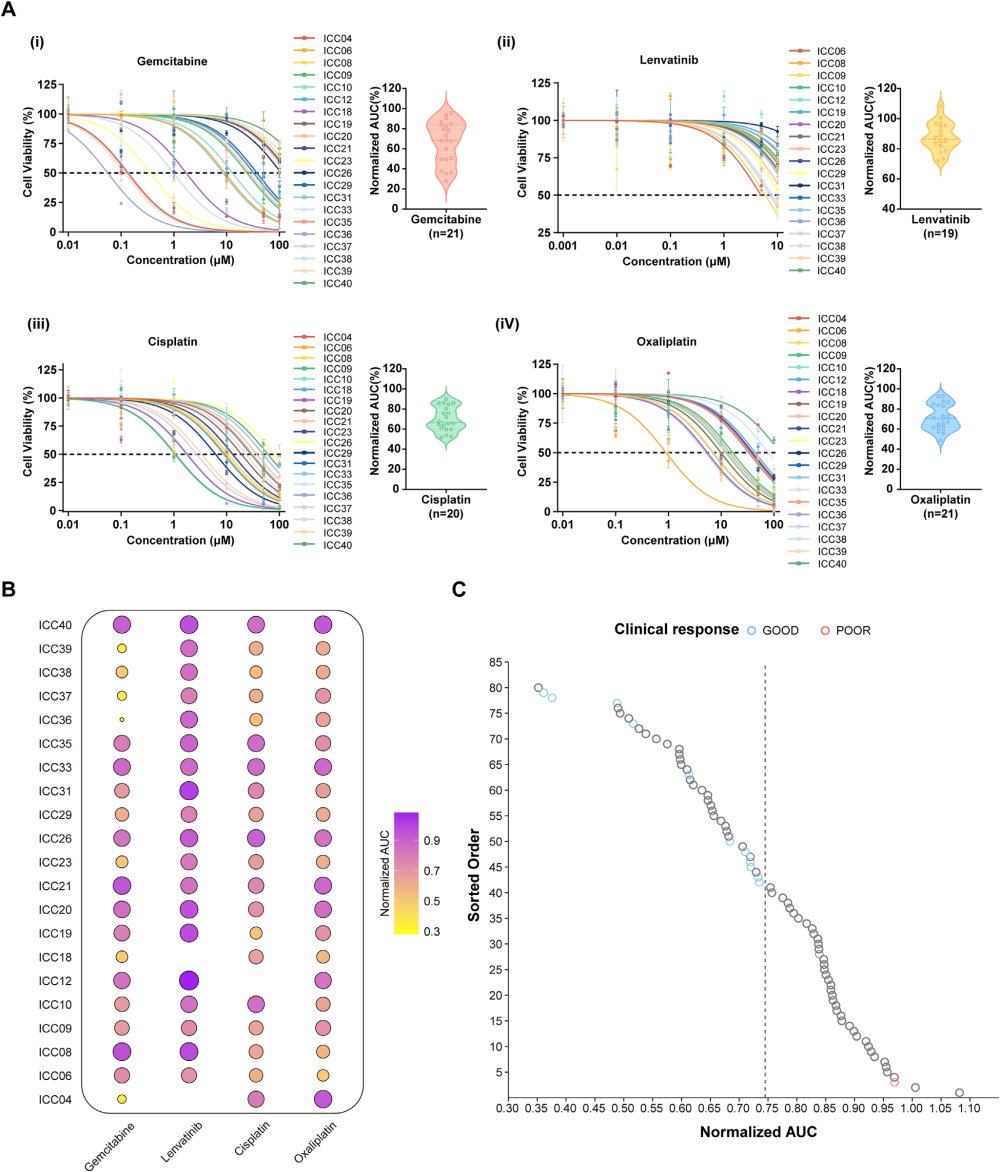
In vitro drug sensitivity profiling of patient‐derived ICC_3DP models and clinical correlations. (A) Pharmacological characterization of ICC_3DP models. Left: dose–response curves illustrating the sensitivity of ICC_3DP constructs to different therapeutic agents. Right: violin plots showing the distribution of normalized area under the curve (AUC) values for four drugs: (i) gemcitabine, (ii) lenvatinib, (iii) cisplatin, and (iv) oxaliplatin. (B) Bubble plot summarizing drug sensitivity profiles across 21 ICC patients. Circle size and color intensity represent relative drug resistance levels (darker/larger = higher resistance). Tested drugs: Gemcitabine, Lenvatinib, Cisplatin, Oxaliplatin. (C) Clinical implications of the drug sensitivity threshold. The Area Under the Curve (AUC) values (circles) are associated with the treatment response in ICC patients. The threshold was determined using the Jenks Natural Breaks algorithm. Blue circles indicate an objective response to sensitive drugs, whereas red circles indicate disease progression with resistant drugs.

We evaluated conventional intrahepatic cholangiocarcinoma chemotherapeutics (gemcitabine, cisplatin, oxaliplatin) and the tyrosine kinase inhibitor lenvatinib using 3D‐bioprinted tumor models from 21 patients, generating dose‐response curves and normalized area‐under‐curve (AUC) metrics (Figure 6A; Figure S7). Notably, the ICC_3DP platform revealed pronounced pharmacodynamic heterogeneity among patient‐derived models. Viability decreased in a concentration‐dependent manner, indicating effective drug penetration and the maintenance of pharmacologic gradients within the three‐dimensional microenvironment. While cisplatin and oxaliplatin exhibited class‐concordant responses in most models, ICC10 demonstrated divergent platinum sensitivity, revealing patient‐specific resistance patterns (Figure 6B). This validated 3D bioprinting approach provides a physiologically relevant, high‐throughput system for precision drug testing and mechanistic studies in ICC.

### 2.7 The ICC_3DP Model Serves as a Potential Tool for Predicting Clinical Drug Sensitivity

We assessed the predictive relevance of in vitro drug screening outcomes for therapeutic responses in ICC. Drug sensitivity testing was performed on samples from 21 patients. For patients ICC04, ICC12, and ICC18, the limited tumor tissue volume restricted cell availability, permitting drug screening with only three drugs per sample. Standardized AUC values were sorted in descending order and categorized into four distinct groups using the Jenks Natural Breaks algorithm with a goodness‐of‐fit exceeding 90%, establishing a resistance cutoff value of 0.735 (Figure 6C; Figure S8). A patient‐derived 3DP model was considered sensitive to a treatment regimen if any drug in the regimen yielded a standardized AUC value below 0.735. Conversely, the 3DP model was classified as resistant only when all drugs in the regimen demonstrated standardized AUC values above this threshold (Table S2). For instance, patients ICC09, ICC23, and ICC29 exhibited sensitivity to all four tested drugs, while ICC08 and ICC10 showed sensitivity to only a subset of agents, and ICC12 demonstrated resistance to all four drugs. These findings suggest that our in vitro model successfully preserves the significant heterogeneity of patient drug responses.

Subsequently, we conducted a comparative analysis between the patients' pre‐ and post‐treatment clinical outcomes and the drug sensitivity results obtained from bioprinted constructs (Table S3). Among the 21 patients, four (ICC06, ICC09, ICC37, and ICC39) received neoadjuvant therapy before surgery. Patient ICC06 initially received surufatinib but progressed after one cycle, suggesting acquired resistance to this regimen. Subsequent treatment with gemcitabine‐oxaliplatin combined with lenvatinib achieved disease stabilization, indicating retained sensitivity to this alternative combination. Notably, ex vivo drug sensitivity testing in our 21‐patient cohort confirmed ICC06's sensitivity to gemcitabine, lenvatinib, and oxaliplatin, mirroring the clinical response observed following this regimen switch. Similarly, patient ICC09 demonstrated clinical correlation during neoadjuvant therapy: three cycles of preoperative GEMOX (gemcitabine‐oxaliplatin) plus lenvatinib yielded a partial response, which corresponded precisely with ex vivo sensitivity testing showing robust response to all three agents. Imaging evaluation demonstrated a clear treatment response in patients ICC37 and ICC39 following neoadjuvant therapy. Patient ICC37 received six cycles of GEMOX, resulting in a reduction of tumor size from 8.6 cm × 7.1 cm at baseline to 6.1 cm × 4.3 cm after treatment. Similarly, patient ICC39 underwent five cycles of GEMOX combined with lenvatinib and sintilimab, with tumor dimensions decreasing from 8.7 cm × 8.5 cm to 5.7 cm × 4.5 cm (Figure S9A).

In parallel, ex vivo drug sensitivity testing using the ICC_3DP platform demonstrated that tumor models derived from both ICC37 and ICC39 were highly sensitive to gemcitabine and oxaliplatin, in agreement with the observed clinical responses (Figure S9B). The concordance between neoadjuvant treatment outcomes and in vitro drug sensitivity results further supports the predictive value of our drug sensitivity testing platform.

After confirming complete concordance between ex vivo drug sensitivity profiles and neoadjuvant treatment responses in four index cases, we prospectively followed all 21 patients who underwent drug sensitivity testing until manuscript submission. Of these, 11 patients received postoperative adjuvant therapy, including gemcitabine plus cisplatin (GC) in two patients, GEMOX in one patient, and Lenvatinib therapy in four patients, with the remaining four patients receiving alternative regimens or undergoing observation. Figure S9C presents the longitudinal follow‐up data and corresponding chemotherapy regimens for all 21 patients in our cohort.

Our study sought to determine patient‐specific drug sensitivities to inform personalized treatment strategies and enhance clinical outcomes. Postoperative treatment analysis showed that patients who received therapies matched to their sensitivity profiles exhibited improved responses. For instance, ICC06 achieved sustained disease control for over six months with in vivo‐sensitive Lenvatinib, and ICC20 derived prolonged benefit from in vivo‐sensitive cisplatin. In contrast, disease progression was observed in patients administered regimens predicted to be resistant, as illustrated by ICC04 and ICC19, both of whom progressed on ex vivo‐resistant Lenvatinib. Remarkably, no patient receiving an adjuvant regimen containing in vivo‐sensitive agents experienced recurrence or disease progression during follow‐up, underscoring the potential clinical value of our drug sensitivity testing platform for personalizing treatment in this population.

While our study demonstrated favorable outcomes in patients receiving sensitivity‐matched therapies, we also observed cases where patients treated with ex vivo‐resistant agents did not experience progression or recurrence during the follow‐up period. For example, ICC35 received adjuvant gemcitabine and cisplatin (both in vitro‐resistant) but remained progression‐free at the short‐term follow‐up of 4 months, potentially attributable to limited surveillance duration. Similarly, ICC12, whose tumor showed ex vivo resistance to lenvatinib and GEMOX, later received afatinib combined with immunotherapy, which was not included in our panel. These treatments may have reduced recurrence risk through alternative mechanisms beyond those captured by our assay.

## 3 Discussion

The prognosis of intrahepatic cholangiocarcinoma remains dismal, with a 5‐year survival rate of only 20%–35% even after surgical resection [1]. Current therapeutic paradigms heavily rely on adjuvant drug regimens; tailoring adjuvant therapies through precision oncology paradigms is critical for optimizing therapeutic outcomes in ICC [46]. Patient‐specific drug selection guided by integrated molecular profiling and clinicopathological characteristics holds substantial potential to improve progression‐free survival and overall therapeutic efficacy. In parallel, 3D bioprinting has emerged as a powerful platform for tumor modeling by enabling the construction of biomimetic architectures that recapitulate key features of the tumor microenvironment. Beyond predicting clinical treatment responses, these engineered models provide a unique opportunity to interrogate cell‐type‐specific functions and interactions within complex tumor ecosystems [47, 48]. In this study, we systematically evaluated GelMA/HAMA‐based bioinks across concentration gradients. The commercial hydrogel formulation (6.5% GelMA/0.5% HAMA) was selected to establish a standardized extracellular matrix environment suitable for primary intrahepatic cholangiocarcinoma cell growth.

Unlike hepatocellular carcinoma, ICC is characterized by an extensive desmoplastic stroma and increased tissue stiffness, which pose unique challenges for primary cell isolation and ex vivo culture. Standard dissociation protocols developed for softer liver tumors often result in low viable cell yield or excessive cell death when applied to ICC tissue. As a result, conventional single‐enzyme digestion or prolonged enzymatic treatment often leads to low cell yield or substantial cellular injury. Under these conditions, an enzymatic strategy that balances effective stromal degradation with gentle tumor cell dissociation is essential. In the present study, the combination of collagenase IV for matrix digestion and dispase for cell separation, together with stepwise and time‐controlled digestion, allowed preferential recovery of viable tumor cell clusters while reducing damage associated with over‐digestion.

In parallel, primary ICC cells exhibited marked sensitivity to mechanical stress during extrusion‐based bioprinting. Adjustment of printing parameters guided by bioink rheological properties was therefore critical. Maintaining the GelMA/HAMA bioink in a semi‐solid state and lowering extrusion pressure helped preserve cell viability without compromising printing accuracy. Collectively, these ICC‐specific technical refinements facilitated early construct stabilization and enabled reproducible model generation from limited patient‐derived material, thereby supporting rapid downstream functional analyses.

When tumor volume permitted, our model achieved a success rate exceeding 90%. Even with limited cell numbers, the models exhibited robust aggregation behavior. During culture, patient‐derived models consistently formed complex 3D spheroids across all specimens. Histological and immunofluorescence analyses further confirmed that the in vitro models largely retained the morphological and molecular pathological characteristics of the original tumor tissues. Excitingly, our model consistently developed polarized, organ‐like structures during in vitro culture. Branching is a manifestation of functionalization, and organoids exhibiting branching behavior have been reported to more accurately recapitulate native tissue compared to their non‐branching counterparts [49]. Notably, in our immunostaining assays of ICC32_3DP and ICC34_3DP, we observed that cholangiocarcinoma‐derived cells progressively self‐organized into liver‐like morphological structures during in vitro culture. Although this phenomenon remains rare under our current culture conditions, it opens a promising avenue for future investigation. Given the shared developmental origin of the liver and bile ducts, current research in the field has largely centered on the directed differentiation of bipotent progenitor cells with both hepatocytic and cholangiocytic potential [50, 51]. We propose that, by temporally modulating growth factors in the culture medium and establishing a suitable extracellular matrix environment, it may be possible to prompt cells to self‐assemble into complex in vitro models that recapitulate both hepatic parenchymal and biliary structures. As demonstrated by our light microscopy and immunofluorescence imaging, the organoids developed branching structures along with asymmetric and compartmentalized morphological features at specific growth stages. As these observations may indicate successful functional recapitulation within our culture system, they establish a robust platform for further mechanistic investigation. Furthermore, whole‐exome sequencing validated that the mutational profiles of our ICC_3DP models closely recapitulated those of the primary tumors, including alterations in key known ICC driver genes. This preservation of genomic features is essential for maintaining the original tumor's biology.

Comparative transcriptomic analyses revealed that ICC_3DP constructs exhibited upregulation of genes associated with extracellular matrix remodeling, tumor proliferation, and invasion, accompanied by enrichment of PI3K/AKT and ECM–receptor interaction pathways relative to the parental tumor tissues. At first glance, these differences may raise concerns regarding potential deviations from the native tumor signaling state. However, we believe that these changes should be interpreted in the context of three‐dimensional ECM‐mediated mechanoadaptation rather than as a loss of tumor identity.

ICC is characterized by a desmoplastic and mechanically active tumor microenvironment, in which tumor cells are continuously exposed to dense extracellular matrices, altered stiffness, and integrin‐dependent adhesion signaling. Transitioning primary ICC cells from ex vivo tissue into a defined 3D ECM‐supported environment inevitably induces early adaptive transcriptional responses related to cell–matrix interactions, cytoskeletal tension, and survival signaling. The observed activation of ECM‐related and PI3K/AKT pathways is therefore consistent with established mechanotransduction responses reported in multiple 3D tumor culture systems and likely reflects partial recapitulation of the fibrotic ICC niche rather than artificial oncogenic overstimulation [52, 53, 54].

Importantly, our longitudinal observations indicate that these transcriptomic changes represent a transient or stabilized adaptive state rather than a progressive or uncontrolled drift. While ECM‐associated pathways were elevated during early culture phases, the ICC_3DP models retained stable morphology, high cell viability, conserved driver gene mutations, and consistent drug response profiles over time. These findings suggest that ICC_3DP constructs reach a phenotypic equilibrium following initial ECM adaptation, supporting their suitability for downstream functional assays. From a bioengineering perspective, we acknowledge an inherent trade‐off between minimizing ECM‐driven activation of proliferative or invasive signaling and preserving sufficient mechanical integrity and biochemical support for long‐term 3D culture. The GelMA/HAMA composite bioink was designed to balance printability, structural stability, and cellular viability, all of which are essential for reproducible drug sensitivity testing. Excessive attenuation of ECM compositions, such as markedly reducing stiffness or adhesion ligand density, may compromise construct fidelity, cell survival, and assay accuracy.

Nevertheless, we recognize that further optimization of bioink composition represents an important future direction. Potential strategies include fine‐tuning GelMA/HAMA ratios, modulating crosslinking density, or incorporating degradable or modular ECM components to decouple structural support from excessive signaling activation. Future studies integrating mechanical tuning with longitudinal multi‐omics profiling will be valuable for refining ECM neutrality while preserving functional performance.

Standardization of patient‐derived in vitro drug testing platforms remains a major challenge, particularly when primary tumor material is limited, and initial cell yields vary substantially between patients. In this context, the potential impact of initial cell density on model establishment and drug sensitivity readouts represents a critical technical consideration for clinical translation. In the present study, we systematically evaluated whether variation in initial bioink cell concentration within the practical range required for 3D bioprinting would influence construct maturation, culture timelines, or downstream drug response assessment.

To validate the robustness of our platform, we performed parallel 3D bioprinting and drug sensitivity testing using bioinks with different initial cell concentrations. We observed highly comparable IC50 and AUC values across these conditions, confirming that the predictive accuracy of the ICC_3DP model is maintained within this concentration range. While minor statistical differences in cell viability were occasionally observed at specific drug dosage points, these likely resulted from nonlinear cytotoxic effects at extreme concentrations or slight variations in baseline metabolic activity. Importantly, these localized discrepancies did not alter the overall dose‐response trends or the final classification of drug sensitivity. Together, these findings suggest that the ICC_3DP platform maintains reliable standardization and drug response fidelity across a clinically realistic range of initial cell concentrations, thereby enhancing its practicality for routine functional drug testing in a precision oncology setting.

While Matrigel provides a biologically permissive extracellular matrix that supports organoid formation, its batch‐to‐batch variability, undefined composition, and limited tunability present challenges for standardization and scalability. Our side‐by‐side comparison in matched patient samples demonstrated that the ICC_3DP model yields drug sensitivity profiles highly concordant with Matrigel controls. This validates that our chemically defined GelMA/HAMA bioink effectively maintains the intrinsic drug response characteristics of primary tumor cells, thereby bridging the gap between standardized biomanufacturing and biological fidelity.

Leveraging the high fidelity of our in vitro models in recapitulating key tumor characteristics, we applied these systems for drug sensitivity testing. Specifically, 3D bioprinting technology was employed to integrate cells with their extracellular microenvironment, enabling automated and controllable generation of standardized tumor models. This approach facilitates reproducible drug screening through the production of homogeneous, precisely patterned minimal functional units. Due to the limited size of the obtained specimens, the yield of viable cells after primary isolation, which included tissue mincing, enzymatic digestion, and filtration, was typically around six million. This restricted cell number consequently limited the range of therapeutic agents that could be evaluated in each assay. As a result, we selected chemotherapy regimens commonly used in advanced intrahepatic cholangiocarcinoma, including the GC and GeMOX protocols, as well as the targeted agent lenvatinib, for our drug sensitivity testing. Drug response testing across 21 patient‐derived models demonstrated dose‐dependent reductions in cell viability. Notably, the two patients who received neoadjuvant therapy showed complete concordance between clinical outcomes (stable disease or partial response) and ex vivo drug sensitivity results. Furthermore, all relapsed patients during follow‐up had been treated with drugs classified as resistant in our assays. These findings collectively indicate that our ICC_3DP platform serves as a potentially valuable tool for precision medicine by enabling patient‐specific therapeutic predictions.

Current methods for establishing tumor biomimetic models include organoids, organ‐on‐a‐chip, xenograft models, and 3D bioprinting technology. Organoids closely recapitulate the original tissue architecture, yet they lack many cellular components of the tumor microenvironment. Additionally, batch‐to‐batch variations in Matrigel remain an unavoidable limitation. Moreover, Matrigel is costly, and the prolonged culture process may lead to a gradual loss of original tissue characteristics over successive passages, potentially compromising drug testing outcomes [48]. Organ‐on‐a‐chip systems can simulate dynamic microenvironments, but their engineered structures remain relatively simplistic and lack complex 3D architecture. PDX models maintain high tumor fidelity, but their extended cultivation timelines and reliance on large‐scale animal sacrifice for high‐throughput drug screening pose significant ethical and cost burdens. 3D bioprinting demonstrates outstanding advantages for drug sensitivity testing. By using primary cells directly isolated from patient tissues and enabling the formation of complex 3D biological structures in vitro, this technology can provide accurate drug response data within just 10 days. 3D bioprinting has gained significant traction in recent years due to its ability to precisely reconstruct in vitro models. Sacrificial printing materials facilitate vascularization and even functional bile duct formation, unlocking new opportunities to mimic the tumor microenvironment and advance mechanistic studies [55, 56, 57, 58].

Moreover, as only a small proportion of postoperative patients received paired therapies in this cohort, the assessment of drug response in this group remains preliminary. Although complete concordance was observed in the four patients who underwent neoadjuvant therapy, larger cohorts and prospective validation are needed to confirm the predictive value of this platform. In this study, we observed consistent sensitivity to platinum‐based chemotherapeutic agents in most patients. However, inter‐individual variations were detected in select cases, demonstrating 3D bioprinting's capability to discern precise efficacy differences among drugs of the same class. These findings warrant further validation through larger patient cohorts. Similarly, while combination drug therapies were not explored in this study due to sample size limitations, 3D bioprinting technology offers clinically valuable potential for performing combination sensitivity analyses, enabling more personalized drug response prediction paradigms for individual patients. Over half of patients exhibited resistance to lenvatinib in our study, potentially attributable to the drug's limited target spectrum. This finding suggests that vascularized tumor models may represent a more physiologically relevant platform for evaluating targeted therapeutics.

A major practical limitation of patient‐derived drug sensitivity testing lies in the limited availability of viable primary tumor cells, which inevitably constrains the number of therapeutic agents that can be evaluated per patient. This challenge is particularly pronounced in ICC, where tumor tissue volume is often restricted and primary cell viability varies substantially across samples.

In this study, the use of automated and mechanically controlled 3D bioprinting enabled the rapid fabrication of a large number of highly uniform in vitro tumor constructs from a small amount of primary tissue. By miniaturizing the printed constructs and precisely controlling bioink deposition parameters and initial cell density, standardized tumor units could be generated within less than 1 h, allowing parallel testing of multiple drugs while minimizing cellular consumption. This strategy substantially improves the information yield obtainable from limited patient‐derived samples, thereby partially mitigating the intrinsic constraints imposed by low cell numbers. Compared with PDX models, which require in vivo engraftment and expansion over several months and therefore rarely align with the clinical decision‐making window of ICC patients, the bioprinted platform enables the generation of actionable drug response profiles within approximately 10 days after surgery. This rapid turnaround is particularly relevant for ICC, given its aggressive clinical course and the limited survival of many patients even after curative‐intent resection.

Primary ICC exhibits pronounced resistance to gemcitabine or cisplatin monotherapy [59]. The combination of these two agents can partially improve therapeutic efficacy and delay the development of resistance, and has therefore been established as the standard first‐line treatment for advanced biliary tract cancer [5]. However, this regimen still fails to achieve satisfactory overall survival and objective response rates in clinical practice. In recent years, multiple clinical studies have further demonstrated that the incorporation of targeted therapy and immunotherapy into conventional first‐line chemotherapy can extend survival in patients with advanced cholangiocarcinoma, highlighting the critical importance of rational combination treatment strategies for improving patient outcomes [1, 4, 60]. Meanwhile, potential synergistic or antagonistic interactions among different drugs can profoundly influence therapeutic responses, tumor recurrence, and patient survival. The high complexity of combination regimens arising from variations in drug classes, dosage combinations, and administration sequences substantially limits the applicability of conventional in vitro models for combination drug sensitivity testing. Standardized in vitro models constructed using 3D bioprinting provide a feasible technological foundation for high‐throughput combination therapy testing. This platform enables the parallel evaluation of multiple drug combinations and dose gradients within the same patient‐derived background, facilitating systematic dissection of synergistic, antagonistic, or redundant drug interactions and thereby aiding in the identification of potentially optimal combination treatment strategies. More importantly, such high‐throughput testing can be completed using limited clinical samples within a clinically relevant time window, offering a practical approach to advancing personalized combination therapy toward early postoperative treatment decision‐making.

## 4 Conclusion

In summary, we established a robust and reproducible in vitro model using commercial‐grade GelMA/HAMA hydrogels with minimal batch effects. Patient‐derived primary cells exhibited favorable proliferation within this platform while retaining critical histopathological and molecular features of parental tumors. Importantly, this model served as an effective personalized drug‐testing platform, demonstrating precise concordance between ex vivo sensitivity results and clinical treatment outcomes. The system's reliability in predicting adjuvant therapy responses provides a translational tool for both high‐throughput drug screening and precision oncology applications.

## 5 Materials and Methods

### 5.1 Participants and Patient Specimens Used for the Research

The human ICC specimens utilized in this study were collected from 35 treatment‐naïve patients who underwent surgical resection at Peking Union Medical College Hospital between July 2024 and December 2025. This study protocol was reviewed and approved by the Institutional Review Board of Peking Union Medical College Hospital (Approval No. I‐25PJ1799) in compliance with the Declaration of Helsinki. Eligible patients met the following inclusion criteria: (1) age ≥18 years; (2) radiographic and clinical diagnosis suggestive of ICC; (3) candidates for curative‐intent surgical resection; and (4) subsequent histopathological confirmation of ICC. Written informed consent was obtained from all participants before tissue collection.

### 5.2 Cell Lines and Cell Culture

The human intrahepatic cholangiocarcinoma cell line RBE (CL‐0191) was purchased from Procell Life Science & Technology Co., Ltd. Cells were cultured in RPMI‐1640 medium (Gibco) supplemented with 10% fetal bovine serum (FBS; Gibco) and 1% penicillin–streptomycin (P/S; Gibco). The cells were maintained at 37°C in a humidified incubator with 5% CO_2_ and passaged every 3 days.

### 5.3 Transportation and Preservation of Patient‐Derived Tissue Specimens

For patient‐derived tumor tissues, relatively high‐viability portions were preferentially excised, while adjacent normal tissues were collected from postoperative specimens as controls. For larger tumor specimens, multiple subsections were allocated for primary cell isolation, whole‐exome sequencing (WES), RNA sequencing, and histopathological analysis. All tissues were preserved in a 4°C storage solution and transported on ice, with strict adherence to a 2 h window for either processing (tissue dissociation/experimental procedures) or transfer to −80°C storage. The storage solution consisted of modified DMEM/F12 (Gibco, Billings, MT, USA) supplemented with 0.2% primocin (InvivoGen, San Diego, CA, USA), 1% antibiotic‐antimycotic (Gibco), and 10 µM Rho‐associated kinase inhibitor Y‐27632 (Sigma–Aldrich, St. Louis, MO, USA).

### 5.4 Optimized Isolation of Primary ICC Cells and 3D Bioprinting

Given the pronounced desmoplastic and collagen‐rich stroma characteristic of ICC, a modified enzymatic dissociation protocol was employed to maximize viable cell yield while minimizing cellular damage. Tumor specimens were first minced into ∼1 mm^3^ fragments and subjected to stepwise enzymatic digestion using a combination of collagenase IV (1.5 mg/mL, Gibco), dispase II (1 mg/mL, Sigma‐Aldrich), and DNase I (0.1 mg/mL, Sigma‐Aldrich). Digestion was performed at 37°C with gentle agitation and intermittent mechanical dissociation. At defined time intervals, released cells were collected and immediately transferred into cold medium to prevent over‐digestion, while the remaining tissue fragments were subjected to additional short digestion cycles. This stepwise strategy allowed early‐released viable cells to be preserved and reduced enzymatic stress on fragile primary ICC cells. The resulting cell suspension was filtered through a 100‐µm cell strainer (Biosharp, San Diego, CA, USA) and centrifuged at 300 × g for 7 min. After red blood cell lysis at 4°C for 5 min, cells were washed and resuspended in Advanced DMEM/F12 for counting and subsequent bioink preparation. To preserve cell viability during extrusion, a biomimetic bioink composed of 6.5% (w/v) GelMA, 0.5% (w/v) HAMA, and 0.125% (w/v) LAP (Engineering For Life) was used. Primary ICC cells were gently mixed with the bioink to achieve a final concentration of 3 × 10^6^–6 × 10^6^ cells/mL, loaded into a 3 mL syringe (BD, Franklin Lakes, NJ, USA) fitted with a 23G needle, and incubated at 4°C for 12 min to allow controlled pre‐gelation.

3D bioprinting was carried out using a SUNP Biotech extrusion‐based bioprinter controlled by BioMaker 2i software. Based on rheological optimization of the GelMA‐HAMA bioink, the nozzle and chamber temperatures were set at 20.5°C and 14.5°C, respectively, to maintain the bioink in a semi‐solid state during printing and to reduce shear stress on encapsulated cells. ICC_3DP constructs were printed as grid structures (5 mm × 5 mm × 0.8 mm) with a layer height of 0.2 mm and a strand width of 0.75 mm at extrusion and printing speeds of 1 and 6 mm^3^/s, respectively. The reduced construct size enabled the generation of multiple standardized models from limited patient‐derived material without altering cell density or structural integrity.

Immediately after printing, constructs were photopolymerized using 405 nm light at an intensity of 20 mW/cm^2^ for 24 s, with LAP serving as the photoinitiator. Printed constructs were cultured in 48‐well plates containing 250 µL of primary ICC medium (Table S4), refreshed every 72 h, and maintained at 37°C in a humidified incubator with 5% CO_2_.

### 5.5 Matrigel‐Based Culture of Patient‐Derived Primary Tumor Cells

For method validation, primary ICC cells from selected patients were divided into two groups. The experimental group was processed for 3D bioprinting as described above. The control group was established using Growth Factor Reduced (GFR) Matrigel (Corning). Briefly, cells were suspended in liquid Matrigel on ice, and 25 µL droplets were plated into pre‐warmed 48‐well plates to form domes. After polymerization at 37°C for 30 min, the same culture medium used for 3D bioprinting was added. Drug sensitivity testing was initiated on day 7 for both groups following identical dosing schedules and viability assay protocols (CellTiter‐Glo 3D, Promega).

### 5.6 Fundamental Mechanics & Rheological Properties of Bioprinting Inks

The rheological properties of the bioink were characterized using a rheometer (MCR 302; Anton Paar) to measure the changes in storage modulus (*G*') and loss modulus (*G*″) under varying shear strains. A precision universal tester (AGS‐X‐50N; Shimadzu) was employed to assess the viscosity changes of the bioink under different shear stresses. All bioink preparation procedures were conducted under light‐protected conditions. Each experiment was performed in triplicate to ensure the accuracy, reproducibility, and reliability of the measurements.

### 5.7 H&E, and Immunofluorescence Staining

For hematoxylin and eosin (H&E) staining, tissues and ICC_3DP constructs were fixed overnight in 4% paraformaldehyde (PFA) (Sigma‐Aldrich). After paraffin embedding, 5 µm‐thick sections were prepared and stained following standard H&E protocols.

Paraffin‐embedded tissue sections were first deparaffinized and rehydrated through sequential treatments with xylene and absolute ethanol. Antigen retrieval was then performed using an EDTA‐based solution (pH 8.0). Subsequently, endogenous peroxidase activity was blocked by incubating the sections in 3% hydrogen peroxide at room temperature for 15 minutes. For CK7 and CK19 cytokeratin staining, simultaneous permeabilization and blocking were performed at room temperature using 0.3% Triton X‐100 (Sigma–Aldrich) and 3% bovine serum albumin (BSA, Sigma‐Aldrich) for 1.5 h. For Ki67 and EpCAM staining, samples were first permeabilized with 0.3% Triton X‐100 for 30 min, followed by three 5‐min PBS washes, and then blocked with 3% BSA for 1 h. The blocked ICC_3DP constructs were incubated overnight with primary antibodies diluted in antibody diluent (Invitrogen), including mouse anti‐CK7 (1:200, Abcam), rabbit anti‐CK19 (1:200, Abcam), mouse anti‐Ki67 (1:200, Cell Signaling Technology), and rabbit anti‐EpCAM (1:200, Abcam). After primary antibody incubation, samples were washed three times with PBS for 10 min each. Subsequently, the samples were incubated with 1:200 diluted goat anti‐mouse 594 (Abcam) and goat anti‐rabbit 488 (Abcam) secondary antibodies for 2 h in the dark, followed by three 10‐min PBS washes. Finally, nuclei were counterstained with antifade mounting medium containing 4',6‐diamidino‐2‐phenylindole (DAPI, Yeasen) for 10 min. The stained cells and sections were imaged using a laser scanning confocal microscope (Nikon A1R, Tokyo, Japan).

### 5.8 Cryo‐SEM

Cryogenic scanning electron microscopy (cryo‐SEM) was performed using a field‐emission scanning electron microscope (Regulus 8220, Hitachi High‐Tech, Japan) equipped with a cryo‐transfer system (PP3010, Quorum Technologies). Fresh samples were gently mounted onto conductive carbon adhesive tabs and rapidly plunge‐frozen in liquid nitrogen slush for 30 s. The frozen samples were then transferred under vacuum to the cryo‐preparation chamber using the cryo‐transfer system. Sublimation was carried out at −90°C for 10 min, followed by sputter coating with gold at a current of 10 mA for 60 s. Subsequently, the samples were transferred into the SEM chamber for observation. Imaging was conducted at a cryo‐stage temperature of −140°C with an accelerating voltage of 5 kV.

### 5.9 Live/Dead Staining and Cell Viability Analysis

Cell viability in the 3DP models was evaluated using Calcein‐AM (CAM; Thermo Fisher Scientific) and Propidium Iodide (PI; Thermo Fisher Scientific). CAM labels live cells with green fluorescence, and PI identifies dead cells with red fluorescence. The models were co‐stained with 1 µmol/L CAM and 2 µmol/L PI at 37°C for 30 min in the dark, and then visualized under a laser scanning confocal microscope (C2/C2si; Nikon, Tokyo, Japan). This staining and imaging procedure was conducted on days 1, 3, 6, 10, and 15 after printing.

For viability quantification, three random fluorescence images were acquired per sample under a 10× objective and exported as uncompressed TIFF files. Image analysis was performed using ImageJ/Fiji software (NIH, USA). CAM (Green) and PI (red) channels were separated and analyzed independently to determine the numbers of live cells (N_live) and dead cells (N_dead), respectively. The final values were averaged from the three fields of view.

Cell viability was calculated using the following formula: CellViability(%)=N_live/(N_live+N_dead)×100%

### 5.10 Drug Testing and Cell Activity Assay

Individualized drug response testing was performed on ICC_3DP models using chemotherapeutic agents commonly employed in intrahepatic cholangiocarcinoma treatment regimens. The tested drugs included gemcitabine (Selleck), oxaliplatin (Selleck), cisplatin (Selleck), and lenvatinib (Selleck). Standardized protocols involved drug administration starting on day 7 of in vitro culture, maintained for 3 consecutive days with daily drug replenishment. Concentration gradients were established as follows: 0, 0.1, 1, 10, 50, and 100 µM for gemcitabine, oxaliplatin, and cisplatin; 0, 0.01, 0.1, 1, 5, and 10 µM for lenvatinib. Post 72 h treatment, cellular viability was quantified using CellTiter‐Glo 3D Cell Viability Assay (Promega, Madison, WI, USA) per manufacturer's protocol, with luminescence measurements acquired via microplate reader (Synergy H1, USA). Dose‐response curves were generated, and logarithmic IC50 values were calculated using GraphPad Prism 10.2.3 (GraphPad Inc., La Jolla, CA, USA).

### 5.11 Whole Exome Sequencing and Analysis

DNA was extracted from ICC tissues and ICC_3DP models following a 10‐day culture period using the DNeasy Blood & Tissue Kit (QIAGEN) per the manufacturer's instructions, with subsequent assessment of quantity, purity, and integrity via Qubit fluorometry, NanoDrop spectrophotometry, and 1% agarose gel electrophoresis, respectively. The DNA was then fragmented using a Covaris M220 Focused‐ultrasonicator for library construction, followed by exome capture with the Agilent SureSelect Human All Exon V6 platform and paired‐end 150 bp sequencing on an Illumina NovaSeq 6000 system, achieving a mean on‐target coverage of 150× for tumor samples and 100× for 3DP models. Bioinformatic analysis involved alignment to the GRCh37/HG19 reference genome using BWA (v0.7.9a), post‐processing with GATK and Picard for realignment, base quality recalibration, and duplicate removal, variant calling of SNVs and indels via GATK HaplotypeCaller (v3.3.0), and CNV detection using FACETS, with final mutation files converted to MAF format by vcf2maf (v1.6.21) and visualized using the R package maftools.

### 5.12 RNA‐seq Analysis

RNA sequencing was performed by Anoroad Gene Technology (Beijing, China). Total RNA was extracted from matched ICC_3DP models and their corresponding parental tumor tissues using standard protocols, and RNA quality and integrity were assessed using an Agilent Bioanalyzer 2100 (Agilent Technologies). Sequencing libraries were prepared using the NEBNext Ultra II RNA Library Prep Kit according to the manufacturer's instructions. After normalization and pooling, libraries were sequenced on an Illumina NovaSeq X Plus platform (Illumina, San Diego, CA, USA) to generate paired‐end reads. Raw sequencing data were processed using a standard Illumina pipeline for image analysis and base calling. Gene expression levels were quantified using RSEM (v1.3.1), and expression values were calculated as raw counts, fragments per kilobase of transcript per million mapped reads (FPKM), and transcripts per million (TPM). Differential expression analysis between ICC_3DP models and matched parental tumor tissues was performed using the DESeq2 R package (v1.46.0) based on raw count data. P values were adjusted for multiple testing, and genes with an adjusted *p* value < 0.05 and |log2 fold change| ≥ 1 were defined as differentially expressed genes. Differentially expressed genes were subsequently subjected to Gene Ontology (GO) and Kyoto Encyclopedia of Genes and Genomes (KEGG) pathway enrichment analyses.

### 5.13 Clinical Information of Patients

The postoperative chemotherapy regimen for patients was selected by the attending physician based on individual patient characteristics. Clinical data were collected retrospectively, with progression‐free survival (PFS) used to evaluate the prognosis of intrahepatic cholangiocarcinoma patients. The PFS measurement period commenced from the second day of systemic therapy, with the occurrence of new lesions or disease progression defined as the endpoint event.

### 5.14 Statistical Analysis

Statistical analysis was performed using GraphPad Prism version 10.2.3 (GraphPad Software Inc., San Diego, CA) and R software. IC50 and area under the curve (AUC) values were analyzed through GraphPad Prism 10.2.3, with normalization achieved by dividing individual AUC values by the maximum AUC value of each concentration range. The standardized AUC values of ICC_3DP models were ranked and classified using the Jenks natural breaks algorithm in R package to determine optimal cutoff points by minimizing intra‐group variance while maximizing inter‐group variance. Inter‐group comparisons were conducted using unpaired two‐tailed Student's t‐tests for two groups, while one‐way analysis of variance (ANOVA) was employed for comparisons among three or more groups. A p‐value <0.05 was considered statistically significant (*p < 0.05; **p < 0.01; ns, not significant).

## Author Contributions

Yuce Lu, Liwei Du, Minghao Sun, and Kai Zhang contributed equally to this work. Yuce Lu: Conceptualization, Writing – original draft, Visualization, Investigation. Liwei Du: Writing – review & editing, Investigation, Visualization. Minghao Sun: Methodology, Formal analysis, Supervision. Kai Zhang: Data curation, Visualization. Jiaxun Dong: Methodology, Formal analysis. Xiyue Liu: Methodology. Jiangang Zhang: Methodology. Huiyu Yang: Methodology. Xiaobo Yang: Resources. Xin Lu: Resources. Yiyao Xu: Resources. Yongchang Zheng: Resources. Lei Zhang: Resources. Haitao Zhao: Resources. Xueshuai Wan: Resources. Xinting Sang: Resources. Shunda Du: Resources. Bao Jin: Resources, Supervision. Mingchang Pang: Project administration. Shangze Jiang: Project administration. Fu Xu: Project administration. Hang Sun: Project administration. Yilei Mao: Writing – review & editing, Supervision. Huayu Yang: Writing – review & editing, Supervision.

## Funding

This work was supported by the National Key Research and Development Program of China (2024YFB4607800) and the National Natural Science Foundation of China (32271470, 82472174).

## Ethics Statement

This study was conducted in accordance with the ethical principles of the Declaration of Helsinki. Ethical approval was granted by the Medical Ethics Committee of Peking Union Medical College Hospital (PUMCH), Chinese Academy of Medical Sciences (No. I‐25PJ1799), and human materials were collected after obtaining informed consent from all participants.

## Conflicts of Interest

The authors declare no conflicts of interest.

## Supporting information




**Supporting File 1**: advs74315‐sup‐0001‐SuppMat.docx.

**Supporting File 2**: advs74315‐sup‐0002‐TableS1.xlsx.

**Supporting File 3**: advs74315‐sup‐0003‐TableS2.xlsx.

**Supporting File 4**: advs74315‐sup‐0004‐TableS3.xlsx.

**Supporting File 5**: advs74315‐sup‐0005‐TableS4.xlsx.

**Supporting File 6**: advs74315‐sup‐0006‐VideoS1.mp4.

**Supporting File 7**: advs74315‐sup‐0007‐VideoS2.mp4.

**Supporting File 8**: advs74315‐sup‐0008‐VideoS3.mp4.

**Supporting File 9**: advs74315‐sup‐0009‐VideoS4.mp4.

**Supporting File 10**: advs74315‐sup‐0010‐VideoS5.mp4.

**Supporting File 11**: advs74315‐sup‐0011‐VideoS6.mp4

**Supporting File 12**: advs74315‐sup‐0012‐VideoS7.mp4.

**Supporting File 13**: advs74315‐sup‐0013‐VideoS8.mp4.

## Data Availability

The data that support the findings of this study are available from the corresponding author upon reasonable request.
