# Supplementary material for: Patient‐Derived 3D‐Bioprinted Intrahepatic Cholangiocarcinoma Models Recapitulate Tumor Autologous Traits and Predict Personalized Adjuvant Therapy
Source: Adv Sci (Weinh). 2026 Feb 8;13(22):e22025. doi: 10.1002/advs.202522025 (PMC13088297; doi:10.1002/advs.202522025)
Supplement: Supplementary file 1 — Supporting File 1: advs74315‐sup‐0001‐SuppMat.docx. [file ADVS-13-e22025-s012.docx]

**Supporting Information**

**Patient-Derived 3D-Bioprinted Intrahepatic Cholangiocarcinoma Models Recapitulate Tumor Autologous Traits and Predict Personalized Adjuvant Therapy**

Yuce Lu^1#^, Liwei Du^1#^, Minghao Sun^1#^, Kai Zhang^1#^, Mingchang Pang^1^, Shangze Jiang^1^, Jiaxun Dong^1^, Xiyue Liu^1^, Bao Jin^1^, Fu Xu^1^, Hang Sun^2^, Jiangang Zhang^3^, Huiyu Yang^4^, Xiaobo Yang^1^, Xin Lu^1^, Yiyao Xu^1^, Haitao Zhao^1^, Shunda Du^1^, Xinting Sang^1^, Yongchang Zheng^1^, Lei Zhang^1^, Xueshuai Wan^1^, Huayu Yang^1*^, Yilei Mao^1*^

^1^ Department of Liver Surgery, Peking Union Medical College (PUMC) Hospital, Peking Union Medical College (PUMC) & Chinese Academy of Medical Sciences (CAMS), Beijing, 100730, China

^2^ Liver Transplantation Center, National Clinical Research Center for Digestive Diseases, Beijing Friendship Hospital, Capital Medical University, Beijing, 100730, China

^3^ Department of Head and Neck Surgery, National Cancer Center/National Clinical Research Center for Cancer/Cancer Hospital, Chinese Academy of Medical Sciences and Peking Union Medical College, Beijing, P. R. China

^4^ Department of Neurosurgery, Xuanwu Hospital, Capital Medical University, Beijing, P.R. China

*** Corresponding author**

**Yilei Mao**

Department of Liver Surgery, Peking Union Medical College (PUMC) Hospital, PUMC & Chinese Academy of Medical Sciences, 1# Shuai-Fu-Yuan, Wang-Fu-Jing, Beijing 100730, China. E-mail: [Pumch-liver@hotmail.com](mailto:Pumch-liver@hotmail.com)

**Huayu Yang**

Department of Liver Surgery, Peking Union Medical College (PUMC) Hospital, PUMC & Chinese Academy of Medical Sciences, 1# Shuai-Fu-Yuan, Wang-Fu-Jing, Beijing, 100730, China. E-mail: [dolphinyahy@hotmail.com](mailto:dolphinyahy@hotmail.com)


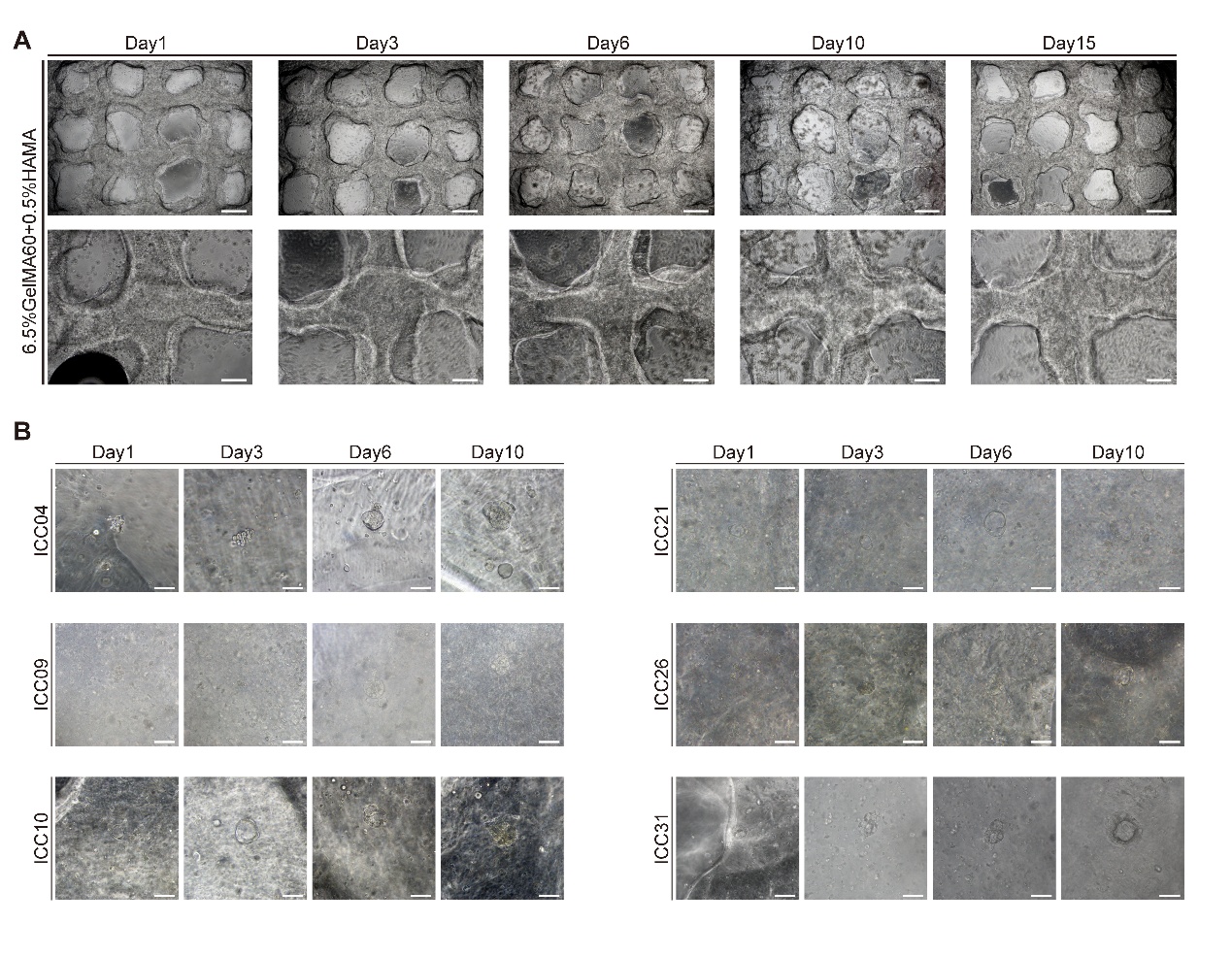


**Figure S1. Stable 3D-bioprinted scaffold maintains structural integrity and supports high viability of patient-derived primary cells. (A)**Structural integrity of 3D-bioprinted RBE-laden GelMA60-HAMA constructs over 15 days (top scale bar: 500 μm; bottom scale bar: 200 μm). **(B)** Bright-field microscopy captures defining morphological features of ICC_3DPs (imaged at Days 1, 3, 6, and 10 post-printing; Scale bar: 50 μm).


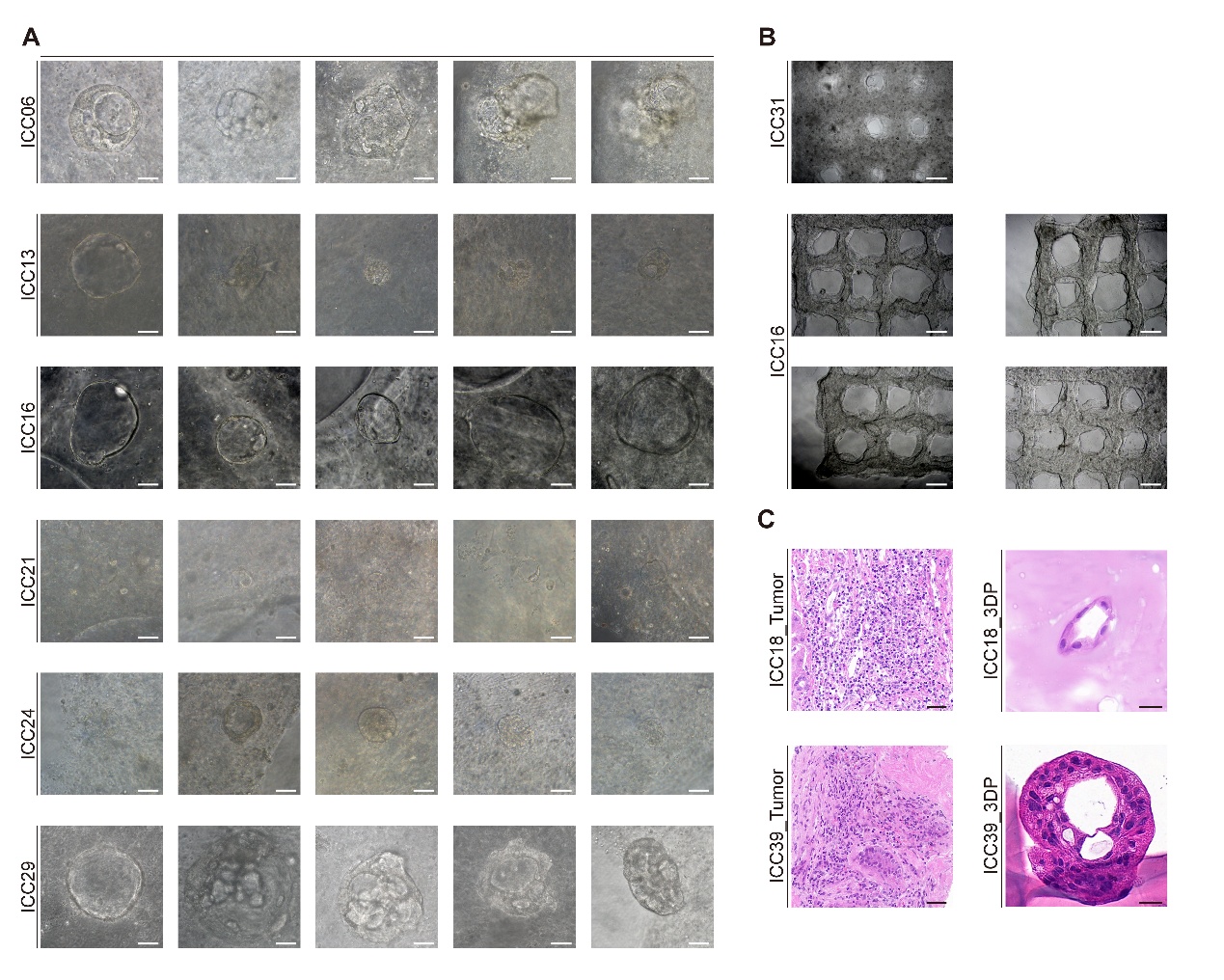


**Figure S2. Patient-derived primary cells exhibited tumor tissue-like morphology within the hydrogel, resembling their parental tumor architecture. (A)**Bright-field images show the spontaneous self-assembly of primary cells into complex, three-dimensional, organoid-like structures within the ICC_3DPs (Scale bar: 50 μm). **(B)** Low-magnification view demonstrating robust cell proliferation within the ICC_3DPs (Scale bar: 500 μm). **(C)**H&E-stained sections of ICC_3DP bioprinted models and their matched primary tumor tissues (left scale bar: 40 μm; right scale bar: 20 μm).


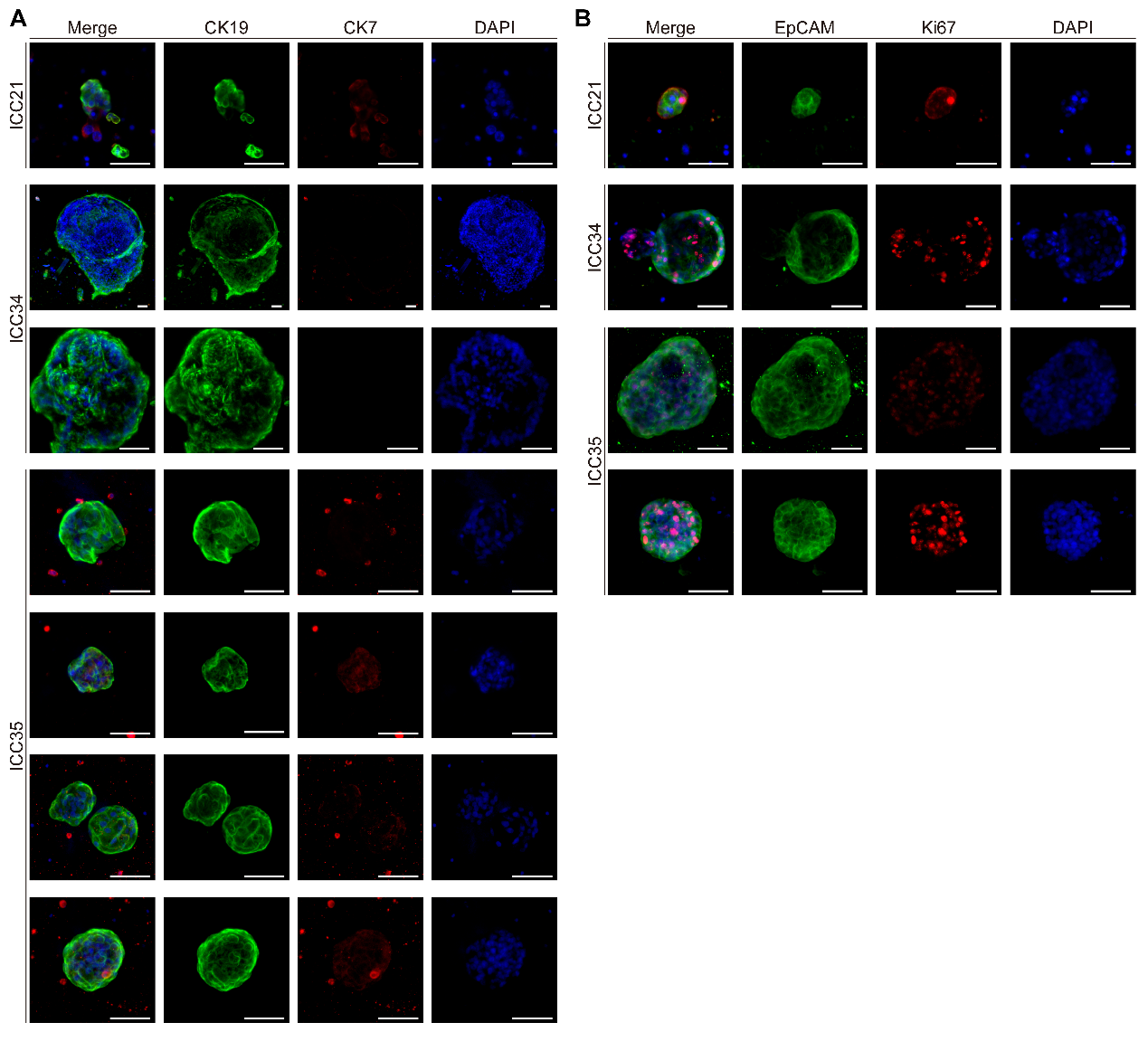


**Figure S3.** **ICC_3DP preserves key molecular markers of intrahepatic cholangiocarcinoma.**

**(A)** Immunofluorescence staining of CK7 and CK19 demonstrated that the ICC_3DP models retained characteristic epithelial markers of the parental tumors. Scale bars: 50 μm. **(B)** Immunofluorescence staining of Ki67 and EpCAM indicated preserved proliferative activity and epithelial cell identity in the ICC_3DP models. Scale bars: 50 μm.


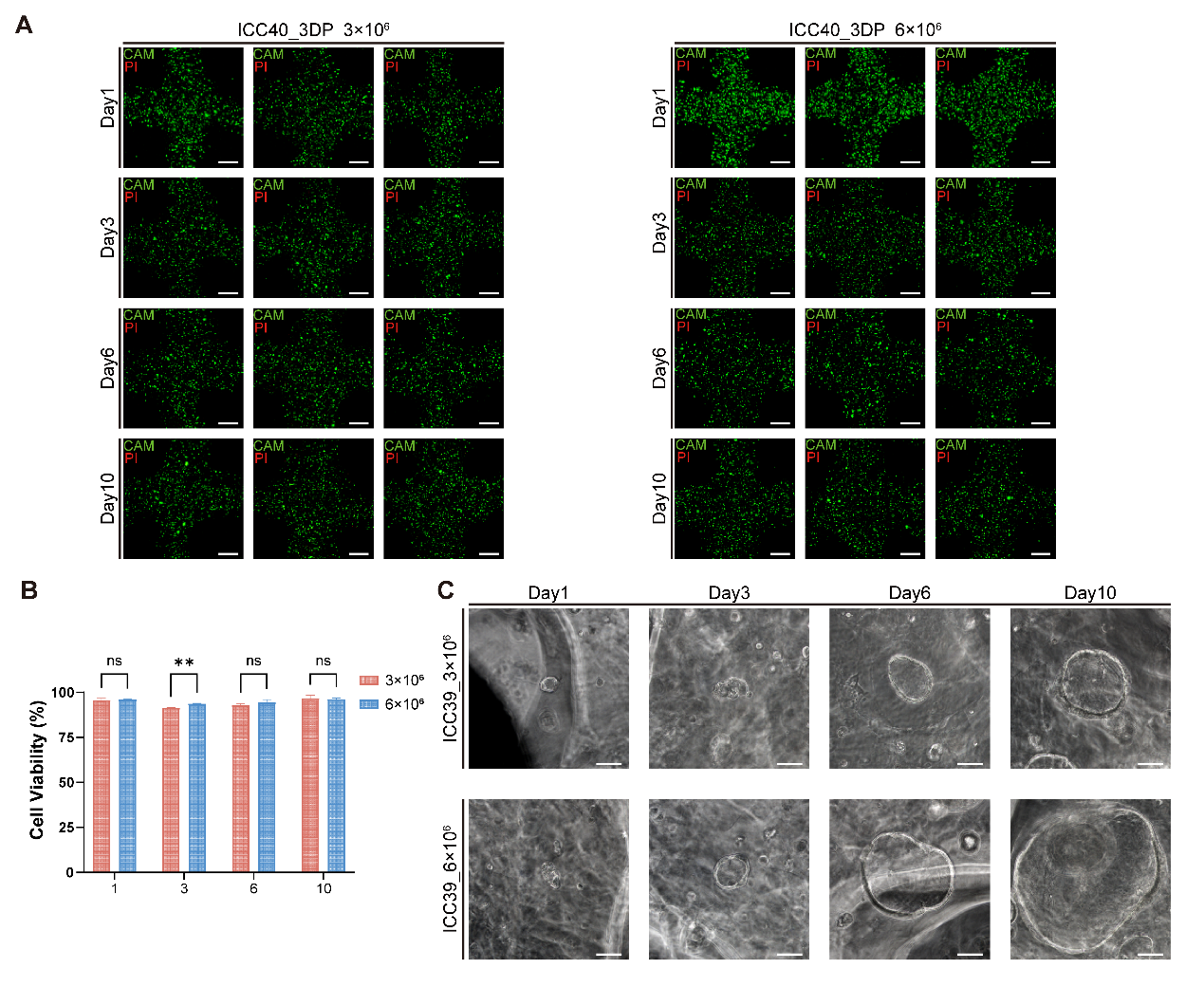


**Figure S4. Cell viability and morphology of bioinks with different initial cell densities.**

**(A)** Live/dead staining of in vitro printed constructs with different initial cell concentrations (3 × 10⁶ and 6 × 10⁶ cells/mL) at days 1, 3, 6, and 10. Live cells are shown in green and dead cells in red. Scale bar, 50 μm. **(B)** Cell viability was quantified using ImageJ at two different initial cell concentrations (n=3). Statistical analysis was performed using an unpaired two-tailed Student’s *t*-test (*p < 0.05; **p < 0.01; ns, not significant). **(C)** Bright-field images of the 3DP constructs prepared with two initial cell concentrations at days 1, 3, 6, and 10 (D1, D3, D6, and D10). Scale bar: 50 μm.


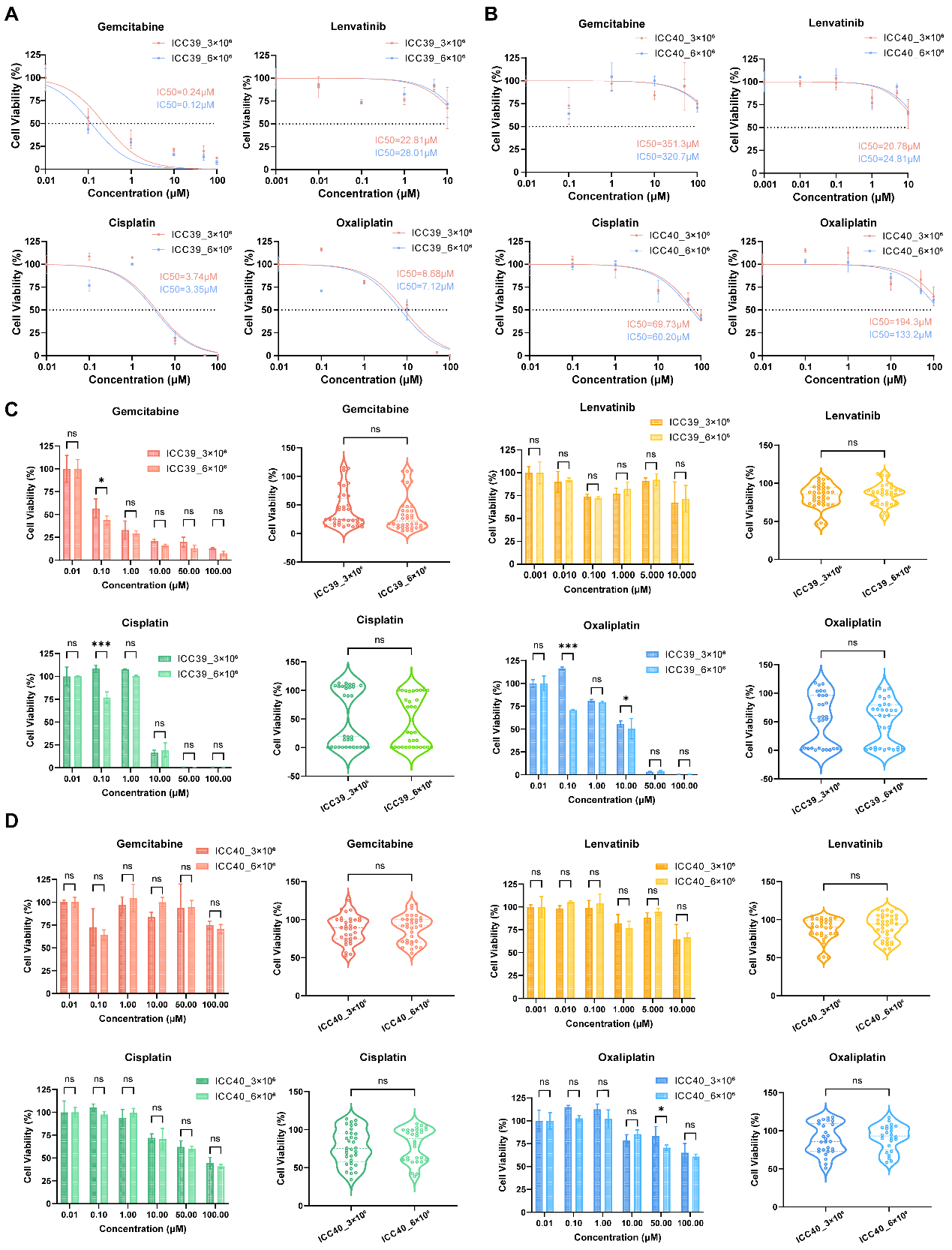


**Figure S5. Different initial cell concentrations on drug sensitivity profiles in patient-derived ICC 3D bioprinted models.**

**(A-B)** Dose-response curves of two patient-derived 3D bioprinted models with two initial cell concentrations (3 × 10⁶ and 6 × 10⁶ cells/mL) following treatment with gemcitabine, lenvatinib, cisplatin, and oxaliplatin.

**(C-D)** Left, bar plots showing cell viability of patient-derived 3D bioprinted models at the indicated drug concentrations, comparing two initial cell concentrations. Right, violin plots showing the distribution of cell viability across all tested drug concentrations for each drug in ICC_3DP models, comparing the two initial cell concentrations. Data are presented as mean ± SD. Statistical significance was assessed using unpaired two-tailed Student’s t-test. ns, not significant; *p < 0.05; **p < 0.01.

**
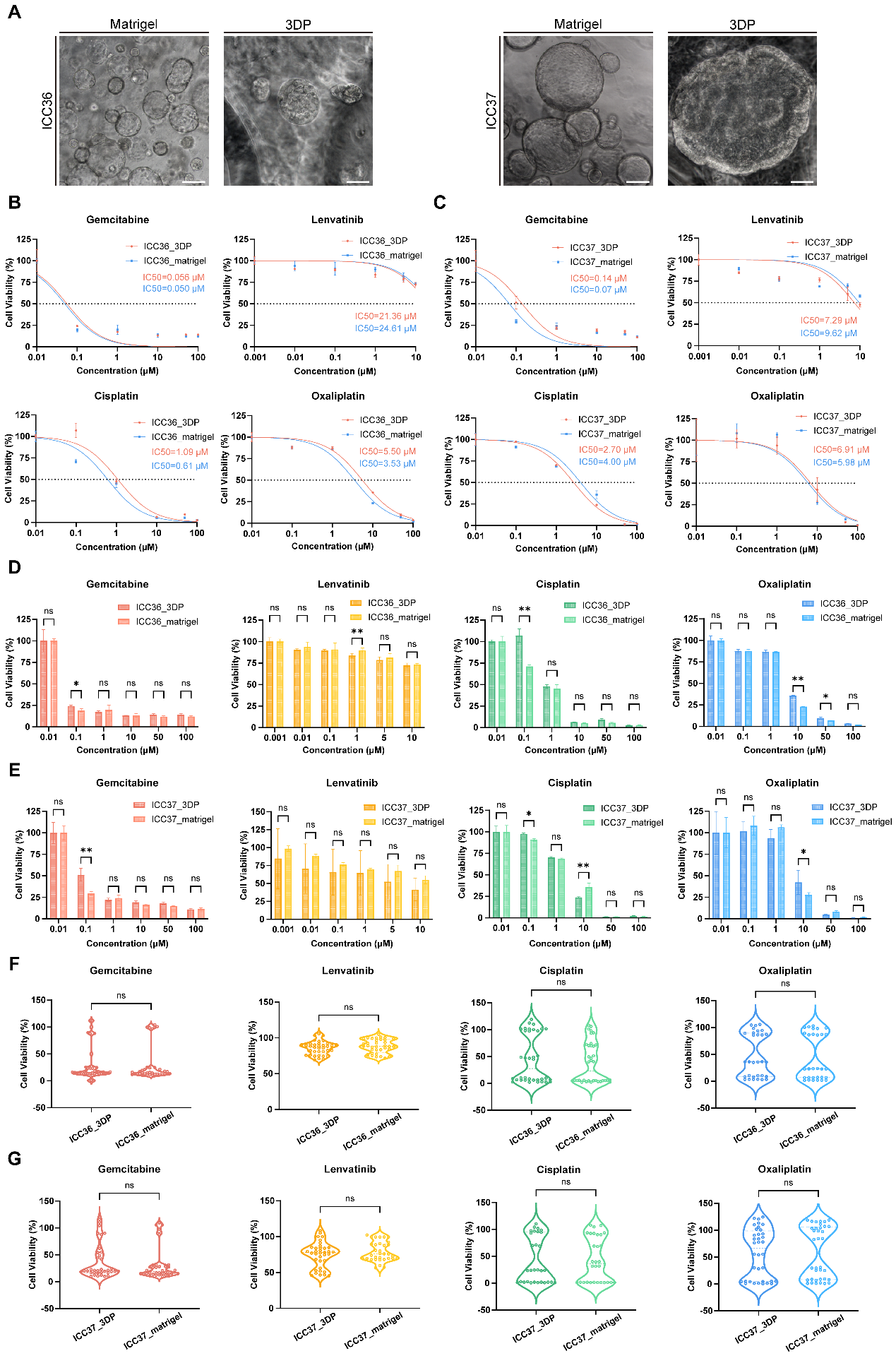
**

**Figure S6. Comparative analysis of drug sensitivity testing using Matrigel-based cultures and 3D bioprinted models.**

**(A)** Representative bright-field images of tumor spheroids derived from two ICC patients (ICC36 and ICC37) cultured using Matrigel-based organoid systems or constructed via 3DP on day 10. Scale bars: 50 μm (Matrigel) and 200 μm (3D bioprinted models).

**(B-C)** Dose–response curves for gemcitabine, lenvatinib, cisplatin, and oxaliplatin in ICC36 (B) and ICC37 (C), comparing Matrigel-cultured organoids and 3D-bioprinted models.

**(D-E)** Bar plots showing cell viability from ICC36 and ICC37 under increasing concentrations of each drug, comparing Matrigel-based cultures and 3D bioprinted models.

**(F-G)** Violin plots showing the distribution of cell viability of ICC36 and ICC37 cultures across all tested drug concentrations, comparing Matrigel-based cultures and 3D bioprinted models. Data are presented as mean ± SD. Statistical significance was assessed using unpaired two-tailed Student’s t-test. ns, not significant; *p < 0.05; **p < 0.01.


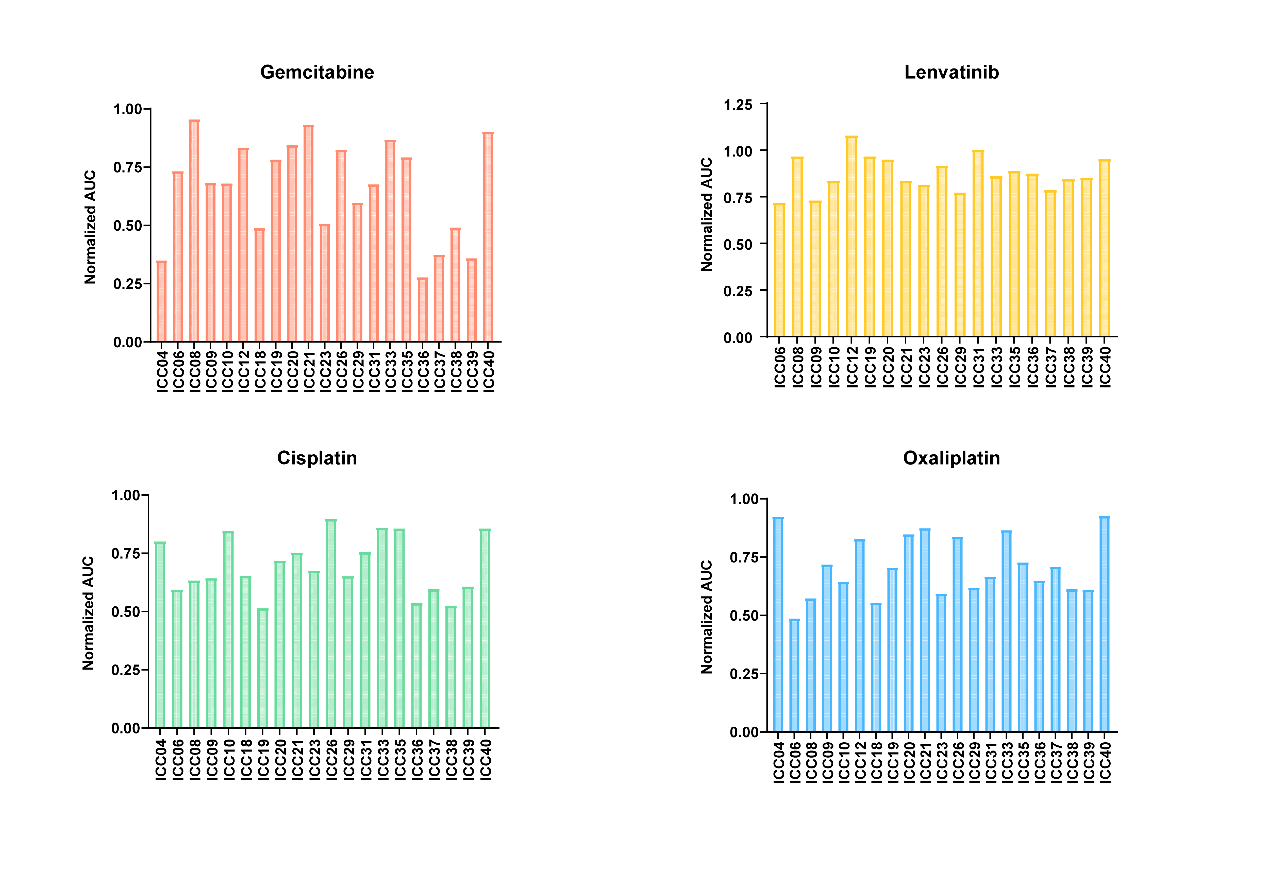


Figure S7. Drug sensitivity profiling of 21 bioprinted ICC models reveals heterogeneous responses to chemotherapy agents (gemcitabine, lenvatinib, cisplatin, oxaliplatin), quantified using normalized area under the dose-response curve (AUC) values.


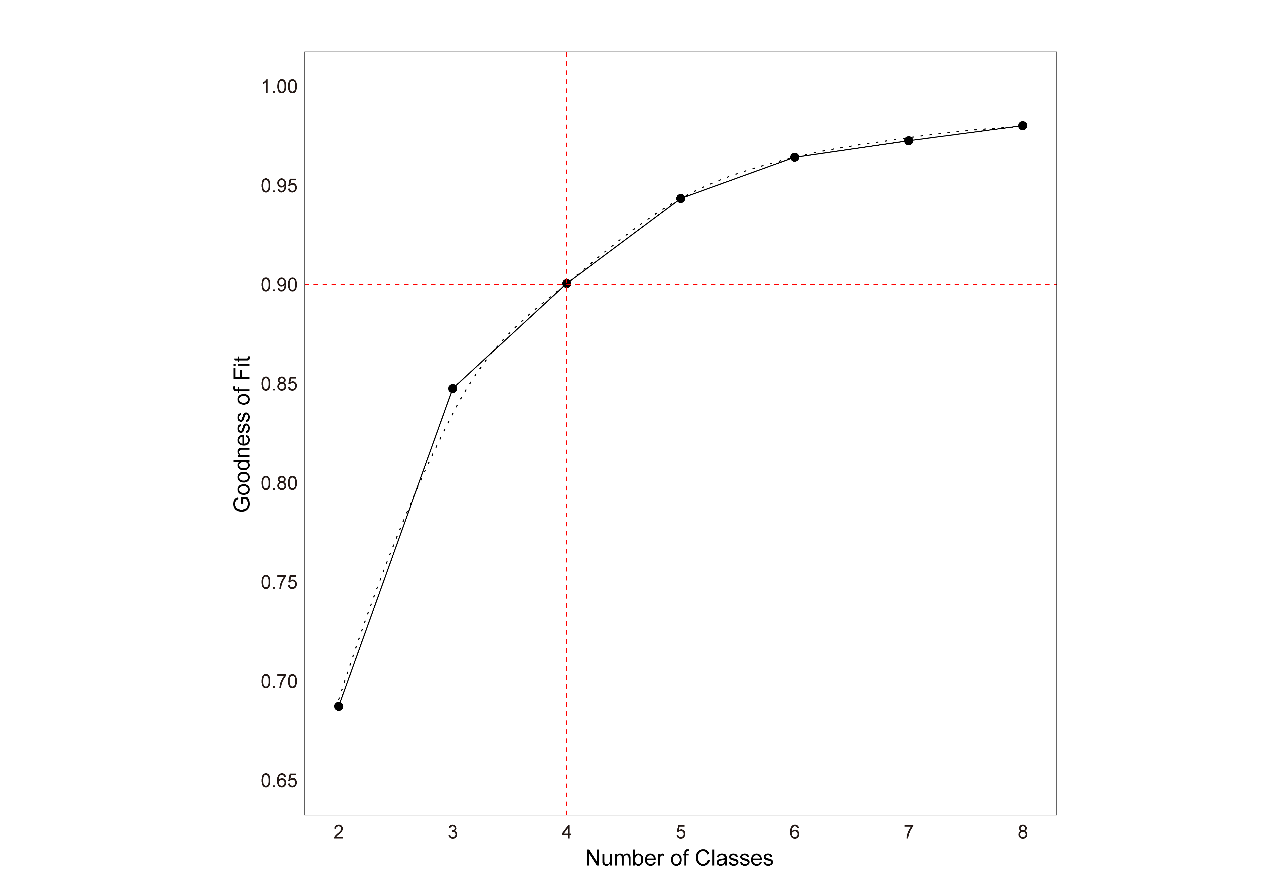


Figure S8. Drug-response heterogeneity classification using Jenks natural breaks optimization (81 AUC values from 21 patient-derived ICC_3DP models). The optimal class number was determined by the minimal categories with goodness-of-fit >0.9 to prevent overfitting.

**
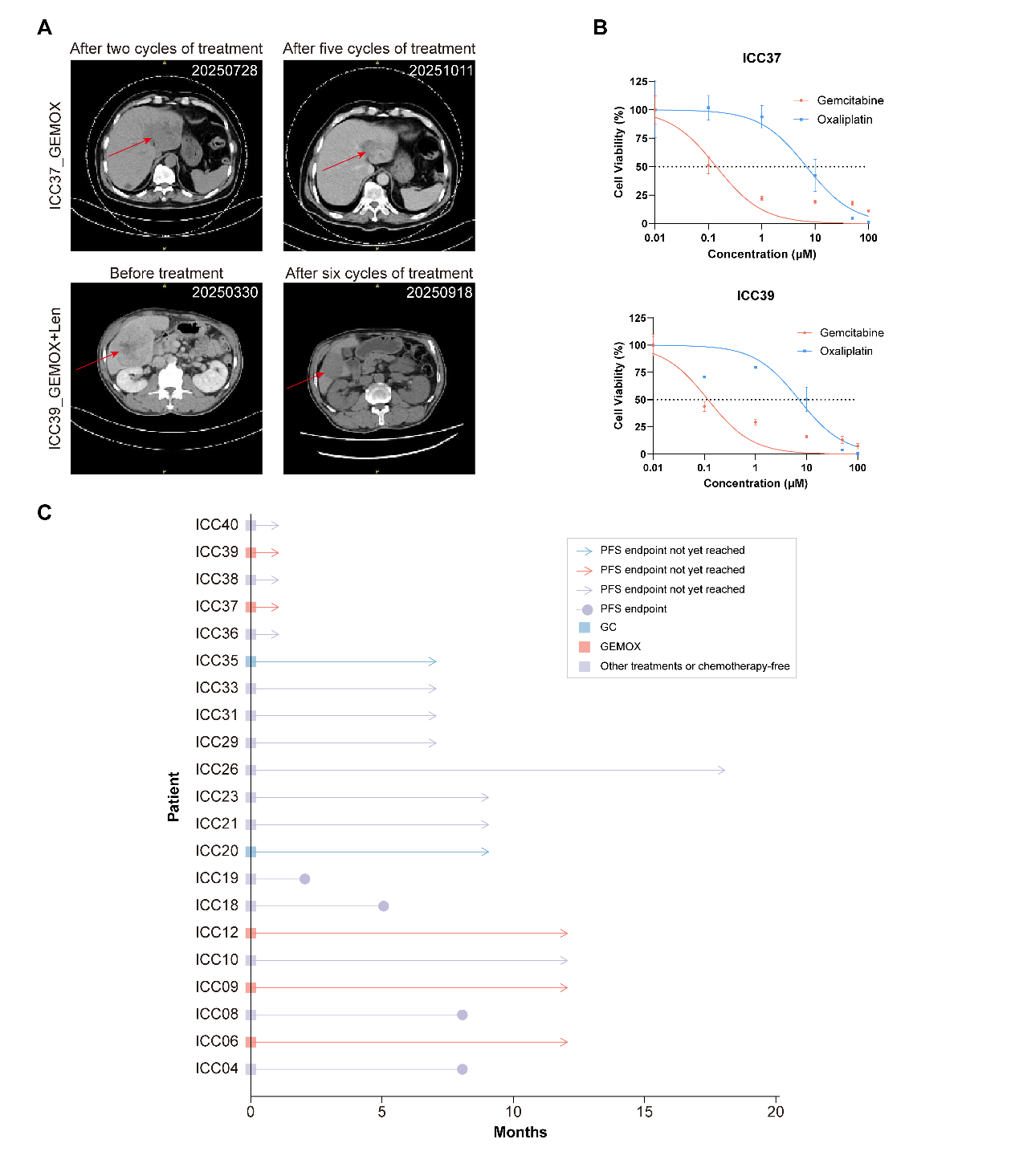
**

**Figure S9. Concordance between clinical therapeutic response and ex vivo drug sensitivity.**(A) CT imaging of ICC37 and ICC39 before and after neoadjuvant therapy demonstrates significant tumor regression (red arrows).

(B) Drug sensitivity curves of corresponding patient-derived 3D-printed ICC models reveal high sensitivity to gemcitabine and oxaliplatin, consistent with clinical outcomes.

(C) Swimlane plot of adjuvant therapies and outcomes in 21 patients with bioprinted, drug-sensitive ICC (ICC_3DP) tumors, revealing associations between treatment pathways and clinical results.
